# Modeling invasion patterns in the glioblastoma battlefield

**DOI:** 10.1371/journal.pcbi.1008632

**Published:** 2021-01-29

**Authors:** Martina Conte, Sergio Casas-Tintò, Juan Soler

**Affiliations:** 1 BCAM - Basque Center for Applied Mathematics, Bilbao, Spain; 2 Department of Molecular, Cellular and Developmental Neurobiology, Instituto Cajal CSIC, Madrid, Spain; 3 Departamento de Matemática Aplicada and Research Unit “Modeling Nature” (MNat), Universidad de Granada, Granada, Spain; Oxford, UNITED KINGDOM

## Abstract

Glioblastoma is the most aggressive tumor of the central nervous system, due to its great infiltration capacity. Understanding the mechanisms that regulate the Glioblastoma invasion front is a major challenge with preeminent potential clinical relevances. In the infiltration front, the key features of tumor dynamics relate to biochemical and biomechanical aspects, which result in the extension of cellular protrusions known as tumor microtubes. The coordination of metalloproteases expression, extracellular matrix degradation, and integrin activity emerges as a leading mechanism that facilitates Glioblastoma expansion and infiltration in uncontaminated brain regions. We propose a novel multidisciplinary approach, based on in vivo experiments in *Drosophila* and mathematical models, that describes the dynamics of active and inactive integrins in relation to matrix metalloprotease concentration and tumor density at the Glioblastoma invasion front. The mathematical model is based on a non-linear system of evolution equations in which the mechanisms leading chemotaxis, haptotaxis, and front dynamics compete with the movement induced by the saturated flux in porous media. This approach is able to capture the relative influences of the involved agents and reproduce the formation of patterns, which drive tumor front evolution. These patterns have the value of providing biomarker information that is related to the direction of the dynamical evolution of the front and based on static measures of proteins in several tumor samples. Furthermore, we consider in our model biomechanical elements, like the tissue porosity, as indicators of the healthy tissue resistance to tumor progression.

## Author summary

Glioblastoma (GB) is a type of brain cancer that originated from glial cells. The infiltrative nature of GB cells is a key feature for understanding its aggressiveness and resistance to current treatments. Cellular protrusions, named as Tumor Microtubes (TMs) in GB, mediate the interaction between tumor and healthy tissue and the processes leading GB invasion. These protrusions are also responsible for several cell communication pathways (e.g. Hedgehog or WNT). We have developed a multidisciplinary approach, which combined biological biomarker measurements performed in *Drosophila* GB with a novel mathematical model, to determine the interactions between proteases, integrins, and TM dynamics. The resulting model is able to predict the formation and infiltration of GB fronts, and, therefore, the directionality, aggressiveness, and progression of the tumor.

## Introduction

Glioblastoma is the most common, aggressive, and lethal tumor of the central nervous system. It has a glial origin and it is characterized by rapid cell proliferation, great infiltration capacity, and neurological impairment [1]. GB is composed of a heterogeneous genetic landscape of tumor cells [2] that reduces the efficiency of clinical treatments. Current treatments for GB include surgical resection of the solid tumor, radiation therapy, and chemotherapy with Temozolomide. However, GB is resistant to treatment and recurrence is the main cause of mortality [3, 4]; the median survival is below 16 months and the incidence is 3/100.000 per year.

GB infiltration in the human brain is a complex phenomenon, influenced by different tumor properties and the tumor microenvironment, including brain-resident cells, blood-brain barrier, and the immune system [5]. GB develops fronts of invasion towards uncontaminated areas of the brain, and GB cells modify their cytoskeleton components [6] to extend protrusions, known as Tumor Microtubes (TMs) [7]. TMs mediate GB progression, and they interact with the synapses of the neighboring neurons [8, 9]. But, how do these fronts occur? Which are the mechanical and chemical processes that characterize these front areas of GB progression? Which are the main agents involved? What is the role of TMs in the tumor front development? Is there any heterogeneity in the spatial distribution of the tumor activity across the tumor domain?

These are some of the questions that we address throughout the paper, providing insights into the role of integrins, metalloproteases, and TMs in GB growth and spread. Our results are supported by experimental measurements in a *Drosophila* model of human GB in continuous feedback with the evolutionary mathematical model, which predicts behaviors and interactions of the biological agents.

### GB in *Drosophila*

The most frequent genetic lesions in GB include the constitutive activation of phosphatidylinositol 3-kinase (PI3K) and Epidermal Growth Factor Receptor (EGFR) pathways, which drive cellular proliferation and tumor malignancy [10]. *Drosophila melanogaster* has emerged as one of the most reliable animal models for GB. It is based on genetic mutations in EGFR and PI3K pathways equivalent to the ones found in patients [11]. Glial cells respond to these oncogenic transformations and reproduce all main features of the disease, including glial expansion and invasion, but in a shorter time. This *Drosophila* model has been used for drug and genetic screenings and the results have been validated in human GB cells [12–14].

### Agents involved in the battlefield

**Fig 1:**
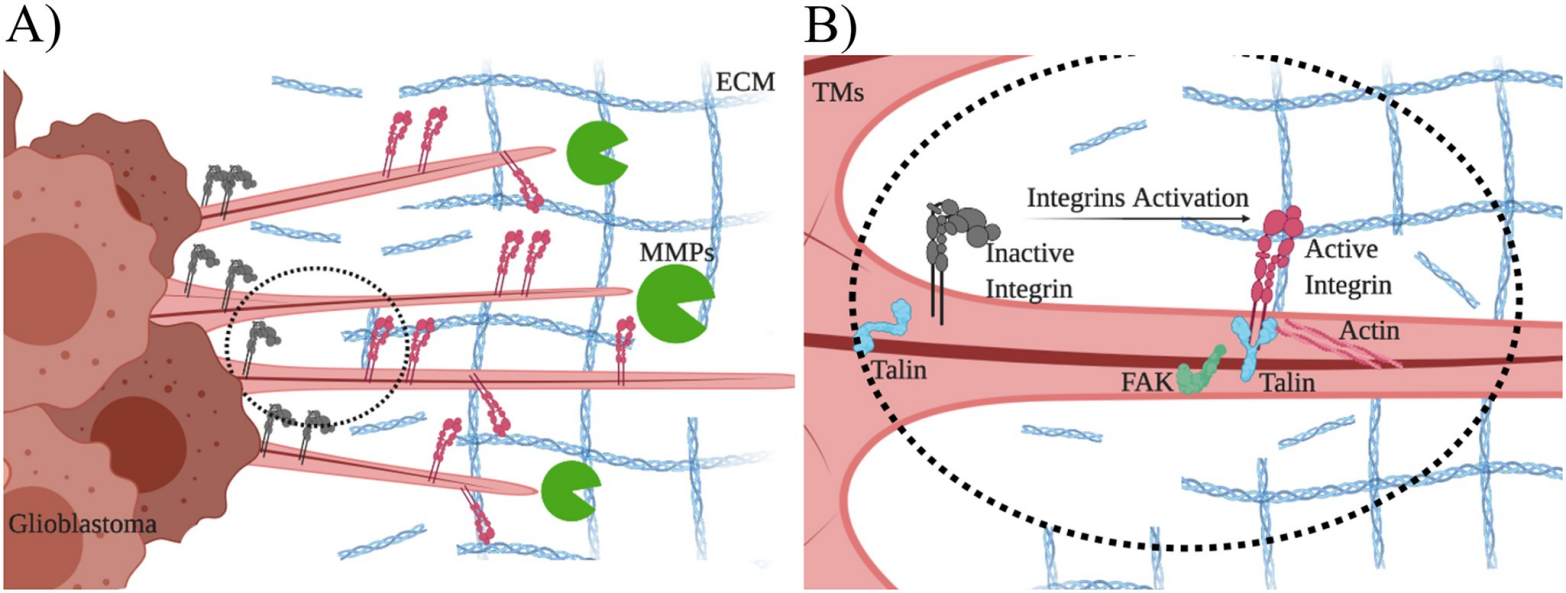
Diagram of GB and protein dynamics. Diagram of the interactions between the proteins involved in GB progression and that give rise to the mathematical model. A): GB cells produce and release in the extracellular space Matrix Metalloproteases (MMPs), which proteolyze the Extra Cellular Matrix (ECM) components. B): Magnification of Tumor Microtubes (TMs). Integrins are activated in the GB tumor microtubes upon interaction with ECM proteins. Active integrins, interacting with Actin filaments and the Talin adaptor protein, activate the Focal Adhesion Kinase (FAK) protein to promote cytoplasm dynamics.

GB growth and migration are driven by specific signaling pathways as well as interactions between the tumor and its extracellular microenvironment. In our study, we include cell membrane protrusions as driving factors of tumor progression, as well as the cell response to signaling gradients and the interplay with the Extra Cellular Matrix (ECM). A schematic diagram of the described dynamics is given in Fig 1.

The dynamics of the tumor cell membrane, including cell protrusions, are fundamental in several processes, among others cell movements, cell transport, and cell exposure to molecular interactions with substrates during GB progression. Generally, cell protrusions are highly dynamic extensions of the plasma membrane involved in cell migration and invasion through the ECM. Different types of protrusions have been identified to contribute to cell spreading, depending on specific contexts, cell types, and microenvironment. Precisely, filopodia are thin, finger-like, and highly dynamic membrane protrusions that have a significant role in mediating intercellular communication and in modulating cell adhesion. These protrusions appear to be required for haptotaxis and chemotaxis [15], i.e., for the cell response to the gradient of insoluble (haptotaxis) and soluble (chemotaxis) components of the tumor microenvironment. Cytonemes are one type of filopodia, first observed in the *Drosophila* wing imaginal disc [16] (see also [17–23]). In addition to the referred intensive studies in *Drosophila*, the importance of cell communication and motility mediated by cytonemes is known in several living systems and it has been largely studied in relation to various signaling pathways in vertebrates (see the seminal paper by Sanders et al [21], the recent results [24–27] and the references therein).

Also known as TMs in the context of GB, cytonemes have an important role in tumor development [12]. Specifically, we focus on TM involvement in the GB front progression and on the relationship of TMs with integrins and proteases.

**Fig 2:**
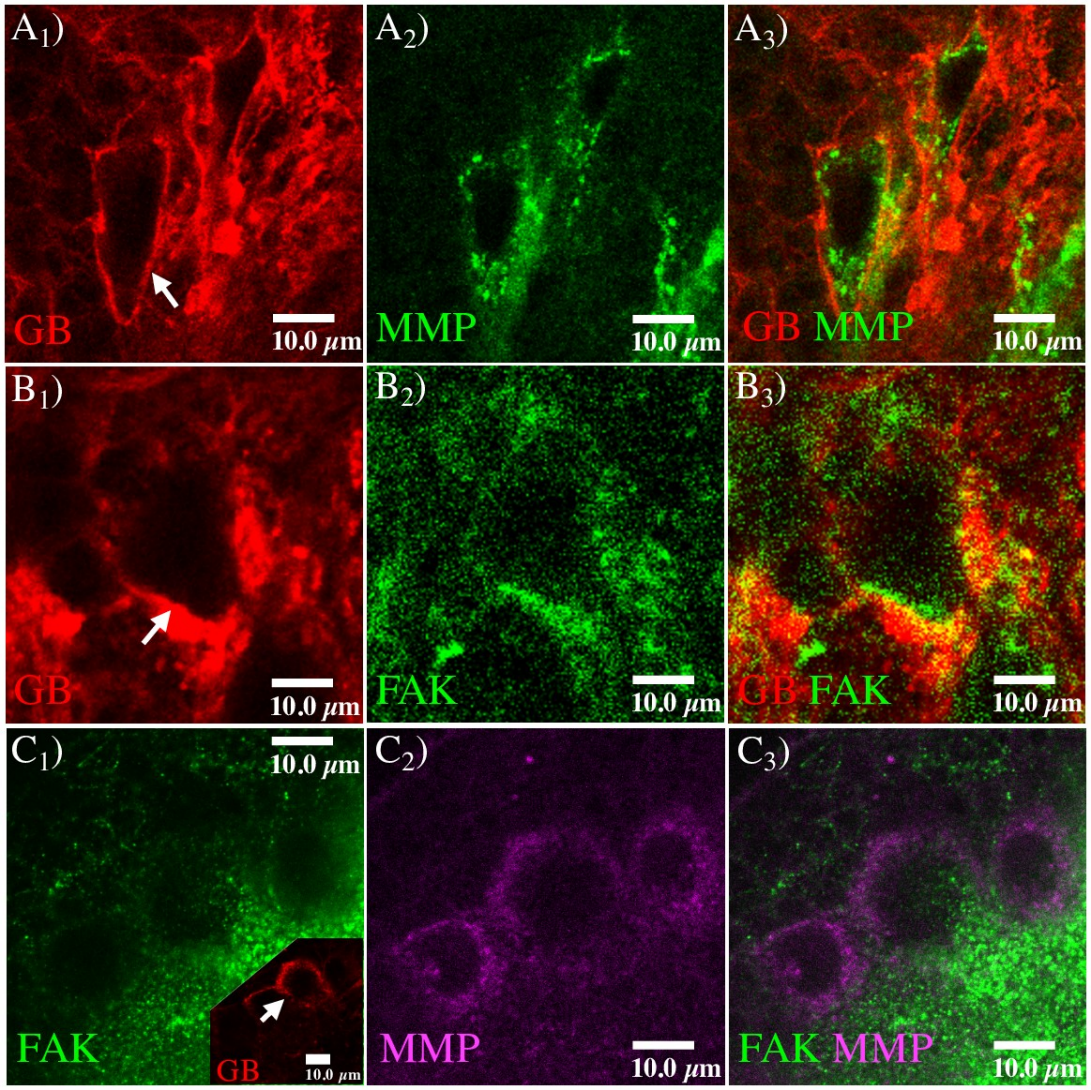
MMP1 and FAK proteins in GB. Fluorescent confocal images of *Drosophila* 3rd instar larvae brain with GB. *A*) Glial membrane is shown in red (*A*_1_), and MMP1 protein in green (*A*_2_). MMP1 accumulation in the GB front is observed in the merged images (*A*_3_). *B*) Glial membrane (*B*_1_, red) and FAK distribution (*B*_2_, green) signals are shown through the brain. FAK accumulation in the GB front is detailed in the merged images (*B*_3_). *C*) Co-staining of FAK (*C*_1_, green) and MMP1 (*C*_2_, magenta), and merged images (*C*_3_). FAK and MMP1 signals accumulate in the same region of GB front (inset in *C*_1_, red). White arrows in the glial membrane images indicate the tumor front, where protein accumulation is observed.

Tumor propagation is characterized by a sharp invasion front located in the front area of GB progression, where TMs are mostly concentrated. The tumor front is a part of the tumor region in contact with the healthy tissue. It is characterized by the presence of TMs/cytonemes and collects a wide activity related to cellular communication signals and cell-ECM interactions. The agents involved in GB invasion, such as integrins, proteases, or the tumor cells themselves, are neither scattered nor randomly moving, but rather there is self-organization that determines invasion patterns around the tumor front. This crucial aspect of the GB progression can be observed in Fig 2.

In this figure, we notice how proteases, identified by the anti-MMP1 (Matrix Metalloprotease 1) antibody, and integrins, identified by anti-FAK (Focal Adhesion Kinase) and anti-Talin antibodies, co-localize with the TM region at the GB front. The prediction of these expansion patterns is a need to envisage possible tumor outcomes in patients and for the development of potential therapies. Therefore, any mathematical model that attempts to predict GB dynamics and reproduce the formation of these evolutionary patterns must face these challenges.

Integrins are transmembrane glycoproteins known to mediate dynamic interactions between the ECM and the actin cytoskeleton involved in cell motility. The binding specificity is determined by the integrin extracellular domain, which allows the recognition of different matrix ligands (fibronectin, collagen, laminin) and other cell surface receptors [28]. In *Drosophila*, the gene *rhea* encodes Talin, a large adaptor protein required for integrin function. Talin links clusters of integrins, bound to the ECM, and the cytoskeleton ant. Therefore, it is essential for the formation of focal adhesion-like clusters of integrins [29]. Integrin activity and cytoskeleton dynamics are mediated by focal adhesion kinase, a cytoplasmic tyrosine kinase driving the cellular adhesion and spreading processes. Integrin interactions with the ECM components support cell migration [30], but affect also cell growth and division through additional interactions with some extracellular proteins and enzymes that control the cell cycle [31, 32]. Integrins are generally present in the cellular membrane, but they are mostly concentrated at the tumor front, where these receptors are directly involved in the signaling leading the migration process.

Proteases are enzymes that catalyze the breakdown of proteins into smaller polypeptides or single amino acids. Some types of proteases are localized around the cell membrane and play a key role in promoting tumor invasion and tissue remodeling. They induce proteolysis of the ECM components [33] and maintain a microenvironment that facilitates tumor cell survival. Protease profiling studies have indicated that the expression of serine proteases, cysteine proteases, and matrix metalloproteases, the most prominent family of proteinases associated with tumorigenesis, increases in high-grade astrocytoma compared with the normal brain. In particular, increased MMP levels and tumor invasiveness in human GBs show a strong correlation [12, 34, 35]. Several studies reported the protease localization in TM membrane [36, 37], especially for MMP-2 and MT1-MMP.

Our interest arises from the analysis of the role of TMs in tumor progression and the effect of different drugs on TM structures and dynamics [38, 39], although we are not modeling drug effects in the present work. The inhibition of TMs leads to a decrease in cell migration and proliferation, with obvious consequences on GB treatment [38–40]. The process of growth and retraction of TMs plays a fundamental role in cell-to-cell communication routing [17, 18, 20–23], as well as it constitutes a transient binding platform for essential proteins that regulate tumor dynamics [41]. Among these proteins, the presence of the microtubule plus end-binding protein EB1 correlates with GB progression and poor survival [40, 42]. This discover has led to a great interest in the development of drugs aimed at affecting end-binding protein expression and, consequently, TM dynamics [38]. Moreover, several experiments have reported the influence of proteases on GB development and the role of several integrin receptors in tumor progression (see [43, 44], S1 and S3 Text and S1 Fig for further details).

### Integration of models and data

The ability of mathematical models to predict and guide biological experiments has gathered a great attention. The number of possible factors involved in GB progression is large and the relevance of each of them is still unknown. Thus, exploiting the advances in microscopy and protein signaling, there is a need to integrate the large amount of provided experimental data into mathematical models.

Different mathematical models describe the dynamics of tumors by linear (Fick’s law) or nonlinear (Darcy’s law) diffusion. Some approaches couple the dynamics for tumor cells and environmental agents involved in its evolution, some others consider them independently (e.g. see [45, 46] and references therein). However, the arising of sharp profiles is not compatible with a movement induced by linear diffusive dynamics, although some mathematical models explored this possibility as a first approach.

We focus on the dynamics of the tumor propagation front and the emergence of the coordination between self-organized collective processes. This coordination is strongly non-linear and the different agent dynamics adapt to each other. The propagation front arises and locates in an area that occupies about 5-7 cell diameters ahead of the tumor main body, where we report an enhancement of the tumor activity and the integrin binding (see Fig 2).

We present here a novel mathematical model that covers specific dynamic aspects of tumor progression. From the modeling side, the novelties of our approach include the description of the dynamics of the tumor front and the link of cell membrane movements with the dynamics of some proteins, their concentrations, and their locations in the TM region. In this model, tumor density is governed by haptotactic and chemotactic processes, induced by MMP and active integrins, as well as by a flux saturated mechanism (see [17, 47, 48] and references therein). The latter allows the incorporation of biological features related to tumor dynamics (i.e., the viscosity of the medium and the speed of propagation) and the definition of sharp invasion profiles [47]. Therefore, this model acquires an excellent predictive capability, both quantitatively and qualitatively, since advanced mathematical concepts are confluent with precise biological experiments. Moreover, the outcomes of the model have partly guided the experiments and this proves the model consistency.

## Results

### Localizing the front of GB

We focus on the dynamics of the active front in GB. Precisely, we analyze the distributions of MMP1, integrins, and FAK and their localization in relation to the tumor membrane density. We hypothesize a spatial heterogeneous activity of tumor cells, in terms of proteolysis and binding receptor activation. This spatial heterogeneity might determine the accumulation of MMP1 and the activation of FAK at the tumor front.

First, we show the presence of MMP1 protein at the active front of GB tissue. We dissected *Drosophila* brain samples with a genetically induced GB (*repo>PI3K; EGFR*). To delimitate the tumor front, we induced the co-expression of a membrane bound (myristoylated form) version of the red fluorescent protein (*UAS-myrRFP*) and, accordingly, all GB cell membranes were marked in red (Fig 2*A*_1_, red). MMP1 protein was detected by a specific monoclonal anti-MMP1 antibody (Fig 2*A*_2_, green). The confocal microscopy images show that the MMP1 signal is heterogeneous through the brain, and stronger in specific regions of the GB front, indicated with the white arrows in Fig 2*A*_1_. Then, we visualized focal adhesions with a specific monoclonal anti-FAK antibody (Fig 2*B*_2_, green). The confocal images show that FAK staining concentrates at the GB front, indicated with the white arrows in Fig 2*B*_1_. Finally, we co-stained for FAK (Fig 2*C*_1_, green) and MMP1 (Fig 2*C*_2_, magenta) proteins, and visualized the active front of GB cells (Fig 2*C*_1_, red inset). The confocal fluorescent images show that MMP1 and FAK signals are visible at the tumor front indicated with the white arrows in inset of Fig 2*C*_1_. These results are compatible with our hypothesis that MMP1 and FAK are characteristics features of the leading edge of migrating GB cells, with a central role in the process of GB expansion. We remark that in the figures visualizing the GB membrane, the healthy tissue is the only component not marked with the immunostaining. Thus it is represented by all the black regions in the images Fig 2*A*_1_ and 2*B*_1_, and the inset of Fig 2*C*_1_.

Mathematically, we model the dynamics of the tumor front in a 1D scenario, i.e., we consider (*t*, *x*) ∈ [0, *T*] × Ω, with *T* > 0 and $\Omega =[0,b_{\Omega }]\subset R$. First, we characterize the TM region (*L*_*TM*_) and the heterogeneity of the tumor activity in the tumor domain using the functional F(N). Defined the tumor support *Sup*(*N*) = [0, *b*_*N*_] ⊆ Ω, the TM region is described as

$$\begin{array}{c} L_{TM}≔\left\{x\in \Omega :\exists \alpha \in \left[0,h_{p}\right]\;\text{such}\;\text{that}\;x-\alpha =b_{N}\right\}\;, \end{array}$$ (1)

where *h*_*p*_ is the maximum length of a microtube, while the functional for the tumor activity is given by

$$\begin{array}{c} F\left(N\right)≔\frac{1}{{\left(N*I_{\left[-h_{p},h_{p}\right]}+\epsilon \right)}^{\alpha_{F}}}\;. \end{array}$$ (2)

Here, * indicates the convolution operator, $I_{\left[-h_{p},h_{p}\right]}$ represents the identity function on the interval of semi-amplitude *h*_*p*_, and *ϵ* and $\alpha_{F}$ are parameters used for modulating the tumor activity. These parameters are incorporated into the model on the base of the experimental results and this novel aspect enables for a better fitting of the biological data. With these basis, the dynamics of the tumor cells density *N*(*t*, *x*) is modeled as:

$$\begin{array}{c} \frac{\partial N}{\partial t}=-\frac{\partial }{\partial x}\;J_{N}\left(N,P,A\right)+P_{N}\left(N\right)\;. \end{array}$$ (3)

The total flux of tumor cells (denoted by $J_{N}\left(N,P,A\right)$) is described by a combination of three main factors. First, the dynamics that $J_{N}$ exerts on cell movements include a saturated flow, which allows to define the movement of a sharp (non-diffusive) profile [47] and to incorporate the experimental data about the propagation front velocity and the porosity of the medium. Then, $J_{N}$ collects information about the cell response to the gradient of soluble and insoluble components of the tumor microenvironment, precisely gradients of MMP1 *P*(*t*, *x*) and active integrins *A*(*t*, *x*). The former degrades the ECM, creating space for the tumor to migrate, while the latter is used to describe the migration toward a gradient of recognized adhesion sites. The proliferation term $P_{N}\left(N\right)$ in (3) simply describes a logistic growth of glioma cells. However, several improvements of this growth term could be taken into account. Major details about the different terms and parameters involved in the glioma cell equation and about the possible improvements of the proliferation term are provided in the S1 Text. The combination of the different fluxes and the proliferation term leads to the following equation governing glioma cell dynamics:

$$\begin{array}{c} \begin{array}{cc} \frac{\partial N}{\partial t}= & \nu_{N}\frac{\partial }{\partial x}\;(\frac{N^{m}}{\sqrt{N^{2}+{\left(\frac{\nu_{N}}{v_{N}}\right)}^{2}{\left|\frac{\partial N}{\partial x}\right|}^{2}}}\frac{\partial N}{\partial x})\\ & -\;\frac{\partial }{\partial x}(N\;\frac{a_{1}}{\sqrt{1+{\left(\frac{\partial P}{\partial x}\right)}^{2}}}\frac{\partial P}{\partial x}+N\;\frac{a_{2}}{\sqrt{1+{\left(\frac{\partial A}{\partial x}\right)}^{2}}}\frac{\partial A}{\partial x})\\ & +a_{3}N(1-\frac{N}{K_{N}}) \end{array} \end{array}$$ (4)

Using this modeling approach, we demonstrate not only the role of proteases (in the specific, of the MMP1 family) and integrins in GB motility, but also their co-localization and spatial distribution with respect to the location of the tumor front. In the specific, this front region is characterized by a higher tumor membrane density compared to the bulk tumor (a region of higher tumor density).

### MMP1s distribution

MMP1s facilitate the tumor invasion process by degrading the extracellular matrix. These proteins are produced by GB cells and released in the extracellular space, mostly in the area around the tumor invasion front. Here, TMs are present in a large number, thus, enhancing the proteolytic activity. Therefore, we propose the localization of MMP1s on TMs and the mathematical modeling of MMP1 dynamics in relation to the tumor front.

**Fig 3:**
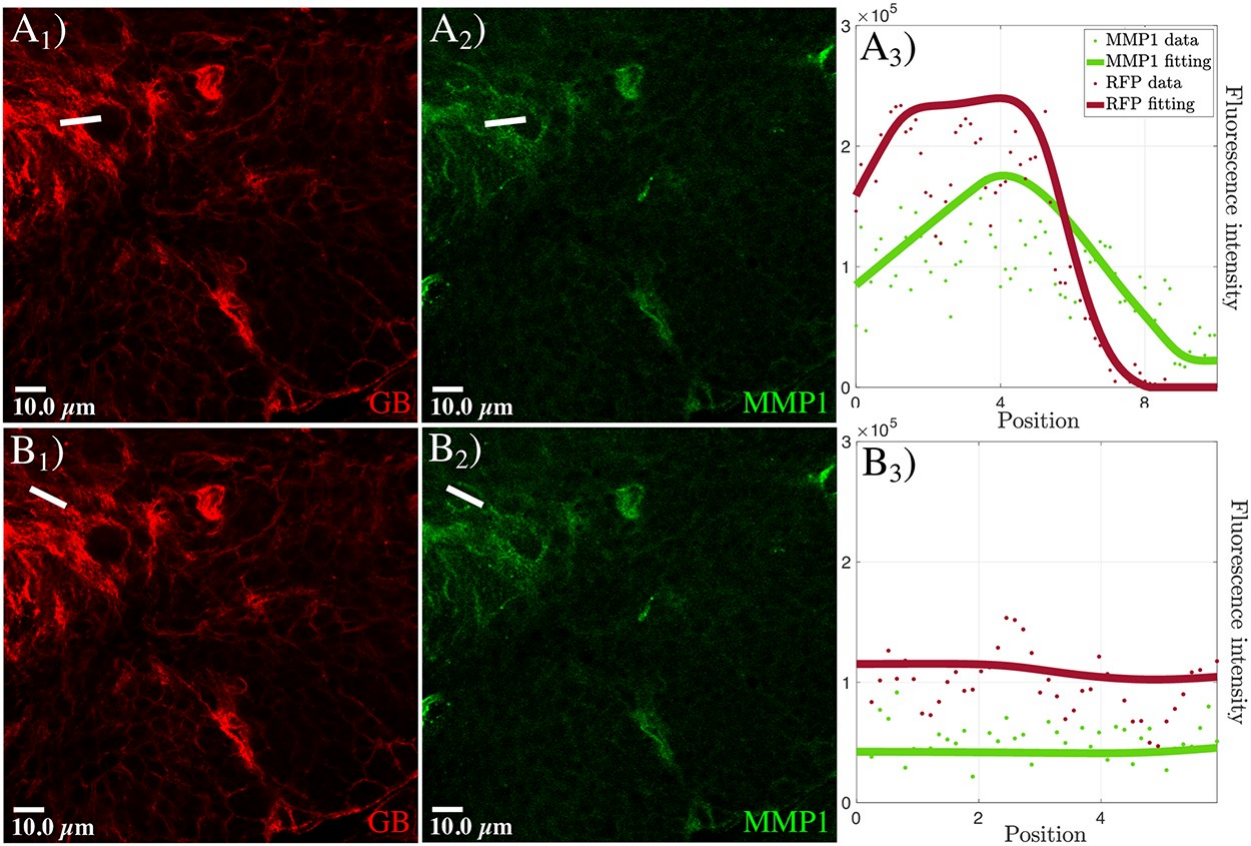
MMP1 accumulation in GB front and inner GB mass. Fluorescent confocal images of *Drosophila* 3rd instar larvae brain with GB marked in red (the white line in *A*_1_ and *B*_1_ indicated the location of the measurement at the front and in the inner GB mass, respectively), and stained with anti-MMP1 in green (*A*_2_ and *B*_2_). In *A*_3_ and *B*_3_, we show the quantification and graphical representations of the fluorescent intensity for GB and MMP1 signals along the white lines in *A*_1_, *A*_2_, *B*_1_ and *B*_2_. Dots represent the data and lines represent the fitting. The healthy tissue is the only component not marked in the immunostaining, thus it is represented by all the black regions in the images *A*_1_ and *B*_1_.

Experimentally, to analyze the protein distribution in the tumor we quantified the MMP1 signal in the inner GB mass and at the GB front. We visualized *Drosophila* brains with GB (Fig 3*A*_1_ and 3*B*_1_) and immunostained with anti-MMP1 (Fig 3*A*_2_ and 3*B*_2_). The results of our analysis indicate that, at the front region of GB (Fig 3*A*_1_) where the membrane density (red curve in Fig 3*A*_3_) is higher, MMP1s (green curve in Fig 3*A*_3_) accumulate, showing a peak of concentration in the region corresponding to the TMs. Interestingly, this MMP1 maximum appears slightly shifted in the direction of GB migration with respect to the peak of GB membrane density. We analyzed the inner GB mass by taking a wider region of GB brains to measure MMP1 (Fig 3*B*_2_) and GB density (Fig 3*B*_1_). The confocal images and their analysis show homogeneous lower levels (basal levels) of GB membrane and MMP1 protein in the inner region (Fig 3*B*_3_) compared to the front (Fig 3*A*_3_). Therefore, these results suggest that MMP1 protein accumulation occurs specifically in the front region of GB, correlating with the peaks of GB cell membrane density in the TM region.

We model the dynamics and the distribution of MMP1s *P*(*t*, *x*) by taking into account three main phenomena: MMP1 production, diffusion in the extracellular space, and degradation. MMP1s are produced by tumor cells and this production is localized along the TM region, where the neoplastic tissue is in contact with the healthy tissue. The production of MMP1 is directly dependent on the heterogeneous tumor proteolytic activity. After MMP1 release into the extracellular space, their flux is limited in the areas surrounding the tumor mass, generating a sharp front ahead of the tumor. In particular, the protease flux is described using the same flux-saturated mechanism used for the tumor cells. In fact, saturated diffusion is not a mechanism exclusive of cells and it can affect species at different scales as long as the differences in size are taken into account in the estimation of velocities and viscosities (further details about tumor cells and proteases related parameters are provided in the S2 Text). Moreover, as the tumor front advances, the remaining MMP1s are degraded and maintain basal levels in the inner tumor region. The overall governing equation for MMP1s is given by:

$$\begin{array}{c} \frac{\partial P}{\partial t}=\nu_{P}\frac{\partial }{\partial x}\;(\frac{P^{m}}{\sqrt{P^{2}+{\left(\frac{\nu_{P}}{v_{P}}\right)}^{2}{\left|\frac{\partial P}{\partial x}\right|}^{2}}}\frac{\partial P}{\partial x})+a_{4}\;E\;F\left(N\right)\chi_{L_{TM}}-a_{5}\;P\;N\;. \end{array}$$ (5)

MMP1 dynamics determines a dynamical degradation of the ECM *E*(*t*, *x*), described as

$$\begin{array}{c} \frac{\partial E}{\partial t}-=-a_{6}\;E\;P\;. \end{array}$$ (6)

We also include a basal level of ECM inside the main tumor mass, as some residual ECM material partially remains in the main tumor region after degradation. Further details about the derivation of the protease and ECM equations and the description of the involved parameters are given in the S1 Text.

### Focal adhesions and integrin dynamics

Integrins are transmembrane receptors that allow cells to bind ECM ligand facilitating tumor cell movement. Upon activation, integrins mediate the organization of the cytoskeleton, cell cycle, and cell migration. In GB cells, the activation process occurs predominantly in the TM region, and active integrins result homogeneously distributed throughout TMs. Moreover, the conversion into the inactive (no bound) state occurs in the proximal region of the TMs with respect to the main GB mass, as shown by our experimental and mathematical results.

**Fig 4:**
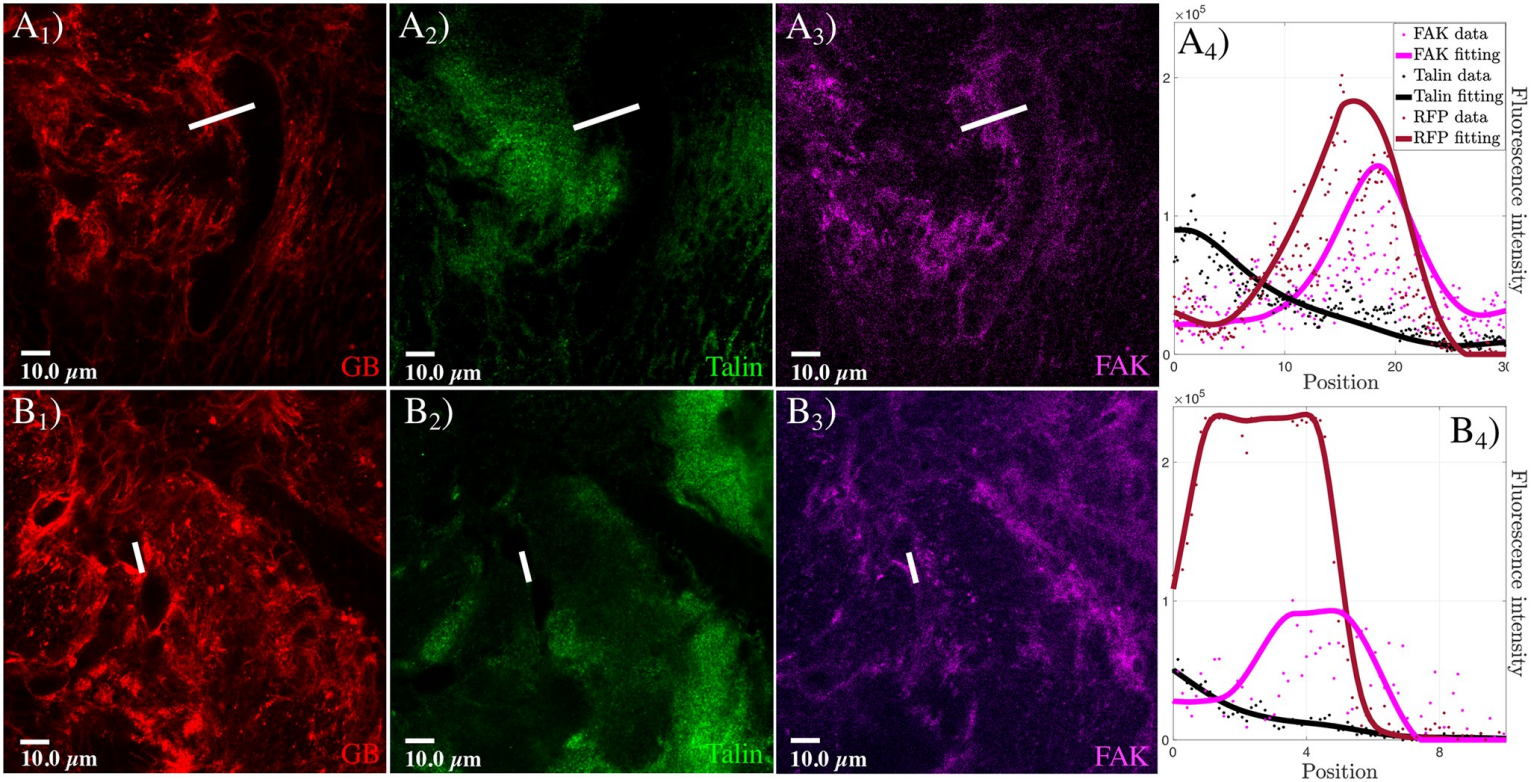
Talin and FAK dynamics in the transition between GB front and healthy tissue. Fluorescent confocal images of *Drosophila* 3rd instar larvae brain with GB marked in red (*A*_1_ and *B*_1_), and stained with anti-Talin (green in *A*_2_ and *B*_2_) and anti-FAK (magenta in *A*_3_ and *B*_3_). In *A*_4_ and *B*_4_, we show the quantification of the fluorescent signals and graphical representation of the fluorescent intensity for GB, Talin, and FAK signals along the white lines in *A*_1_-*A*_3_ and *B*_1_-*B*_3_ that indicate the location of the measurements. Dots represent the data and lines represent the fitting. The healthy tissue is the only component not marked in the immunostaining, thus it is represented by all the black regions in the images *A*_1_ and *B*_1_.

To determine the molecular changes at the GB front in relation to the activity of integrins, we immunostained GB brain samples with Talin, a mediator of integrin adhesivity [49], and with focal adhesion kinase, which is involved in signaling and cytoskeleton dynamics associated with integrin activity [50, 51]. We analyzed confocal microscopy images of GB front regions, and compared fronts with low or high GB membrane signal. The quantification of the signals for anti-Talin and anti-FAK (Fig 4) shows that Talin (black curve in Fig 4*A*_4_ and 4*B*_4_) is reduced in the TM region, i.e., where the GB membrane signal is high (red curve in Fig 4*A*_4_ and 4*B*_4_), while FAK (magenta curve in Fig 4*A*_4_ and 4*B*_4_) is increased. Thus, the pattern of Talin and FAK expression is inverted at the front. Additionally, both the GB membrane and the FAK signals correlate. The GB/FAK signal correlation is maintained in different fronts, irrespective of their low (Fig 4*A*) or high (Fig 4*B*) levels of GB membrane signal. These results suggest an equivalent correlation for the reduction of Talin and the increase of FAK signals at the GB front, consistent with the relation of integrin dynamics and cell motility.

To confirm the functional contribution of integrins to GB progression and to validate our suggestions, we used specific RNAi constructs to knockdown *myospheroid (mys)*, the *Drosophila* Integrin B subunit, or *rhea*, the *Drosophila* Talin, two key players for integrin function. The data show that GB cells require integrins to progress and expand (S1A–S1C Fig). Moreover, *mys* or *rhea* knockdown rescues the lethality caused by GB (S1D Fig). Finally, to confirm Talin and FAK inverse correlation, we compared inner GB mass and GB front areas. The data (S2 Fig) confirm that Talin and FAK maintain an inverse correlation, and they can be consider as indicators of the migratory status of the GB cells (see S3 Text for further details).

Mathematically, we split the integrin population into two subpopulations, referring to the active *A*(*t*, *x*) and the inactive state *I*(*t*, *x*) of the integrin receptors. Their dynamics take into account these processes of activation, and consequent inactivation, as well as a flux term describing the transport process of integrins due to the internal movement of GB cells. Activation is mediated by the presence of the ECM, to which cells bind, and depends on the heterogeneous tumor activity along the TM region. The inactivation of integrins happens once the tumor has crawled on the ECM and moved forward. Since integrin receptors are located on the cell membrane and due to the movement of tumor cells, during the process of cell migration the receptors themselves are also transported, determining a flux of active and inactive integrins with estimated velocity (*v*_*Int*_) depending on the tumor one. Precisely, since we model the dynamics of the bulk tumor, these transport terms allow us to translate the tumor dynamics to the front, where the interaction between integrins and the ECM occurs. Finally, assuming that, initially, the inactive integrins along the cell membrane have not reached their saturation value, a process of integrin production by exocytosis is also included in the model. The equations governing active and inactive integrins dynamics read

$$\begin{array}{c} \begin{array}{cc} & \frac{\partial A}{\partial t}=a_{7}\;E\;I\;F\left(N\right)-a_{8}\;A\chi_{Sup(N)}+v_{Int}\;\frac{\partial A}{\partial x}\\ & \frac{\partial I}{\partial t}=-a_{7}\;E\;I\;F\left(N\right)+a_{8}\;A\;\chi_{Sup(N)}+v_{Int}\;\frac{\partial I}{\partial x}+a_{9}\;\left(K_{I}-A-I\right)\left(\chi_{L_{TM}}+\chi_{Sup(N)}\right)\;. \end{array} \end{array}$$ (7)

Considering the experimental results shown in the S2 Fig, a basal level of active integrins in the tumor main body is included, as well as a basal level of inactive integrins along the TMs. A detailed description of these equations is provided in the S1 Text.

### Motility features of GB cells at the front

The results shown in Fig 2, regarding the features that characterize the tumor front, together with the data on MMP1 and integrin dynamics, support our hypothesis about the processes that lead GB motility.

**Fig 5:**
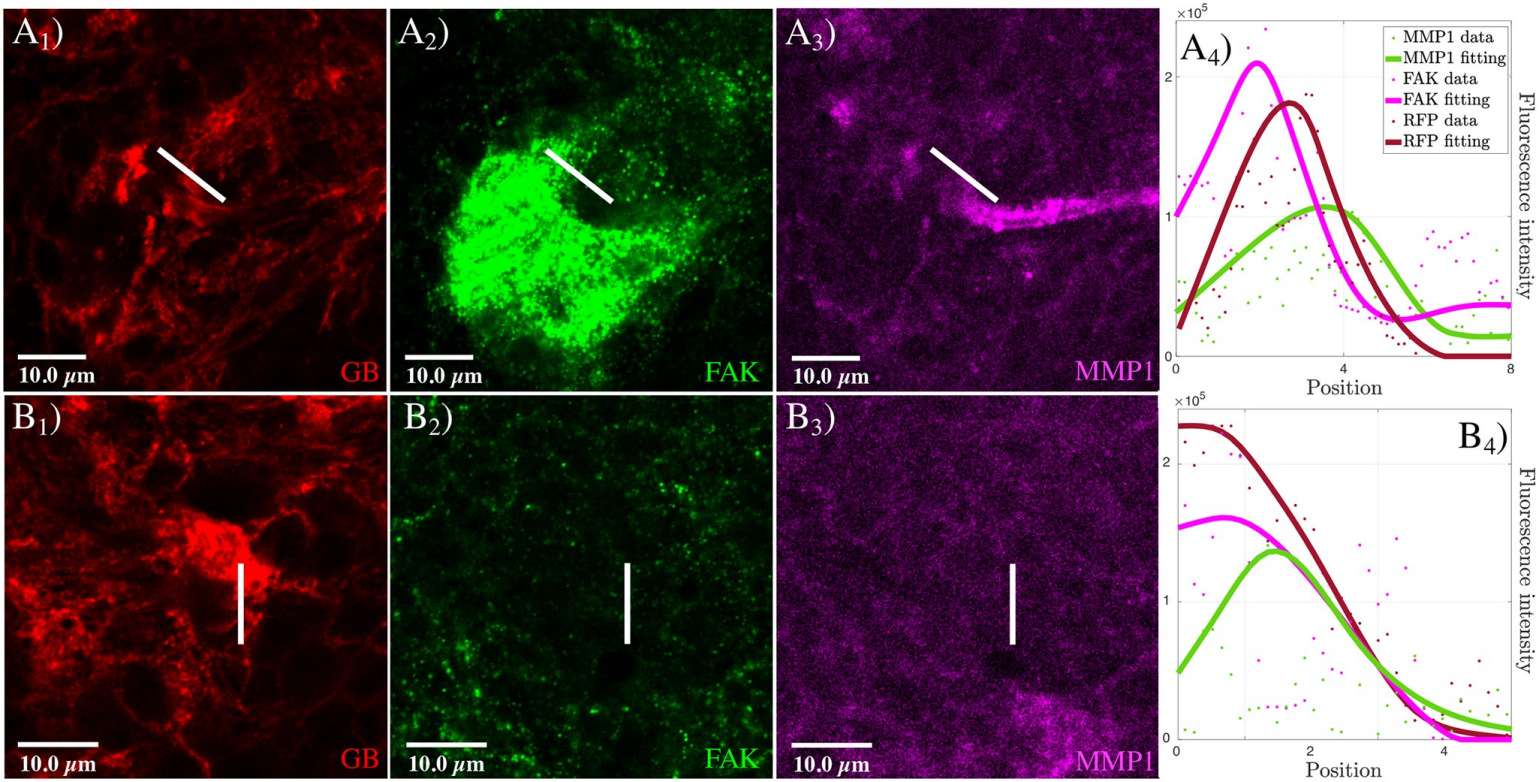
FAK and MMP dynamics in the transition between GB front and healthy tissue. Fluorescent confocal images of *Drosophila* 3rd instar larvae brain with GB marked in red (*A*_1_ and *B*_1_), and stained with anti-FAK (green in *A*_2_ and *B*_2_) and anti-MMP1 (magenta in *A*_3_ and *B*_3_). In *A*_4_ and *B*_4_, we show the quantification of the fluorescent signals and the graphical representation of the fluorescent intensity for GB, FAK, and MMP1 signals along the white lines in *A*_1_-*A*_3_ and *B*_1_-*B*_3_ that indicate the location of the measurements. Dots represent the data and lines represent the fitting. The healthy tissue is the only component not marked in the immunostaining, thus it is represented by all the black regions in the images *A*_1_ and *B*_1_.

Experimentally, we analyzed MMP1 and FAK concentration to prove their role as cues for cell motility, invasiveness, and migration [52, 53]. We co-stained GB brain samples with anti-MMP1 and anti-FAK (Fig 5) and quantified the intensity of the fluorescent signals. We compared the signal in low and high tumor density fronts (Fig 5*A* and 5*B*, respectively). The results of the analysis are shown in Fig 5*A*_4_ and 5*B*_4_ and illustrate that, at the GB front (Fig 5*A*_1_ and 5*B*_1_), the maximum signal for FAK (Fig 5*A*_2_ and 5*B*_2_) occurs before the peak of MMP1 (Fig 5*A*_3_ and 5*B*_3_). This correlation occurs for both types of GB fronts, suggesting that this phenomenon is not a consequence of higher or lower GB membrane density. These results point towards a coordinated function between MMP1 activity and FAK dynamics and suggest that, at the front, FAK acts closer than MMP1 to the GB mass. By contrast, MMP1 plays its role further away from the front. Mechanistically, these results indicate that MMP1 activity in the proteolysis of the ECM is prior to the increase of integrin dynamics, it is associated with the presence of FAK, and, thus, together they contribute to the motility of GB cells.

### Modeling results

**Fig 6:**
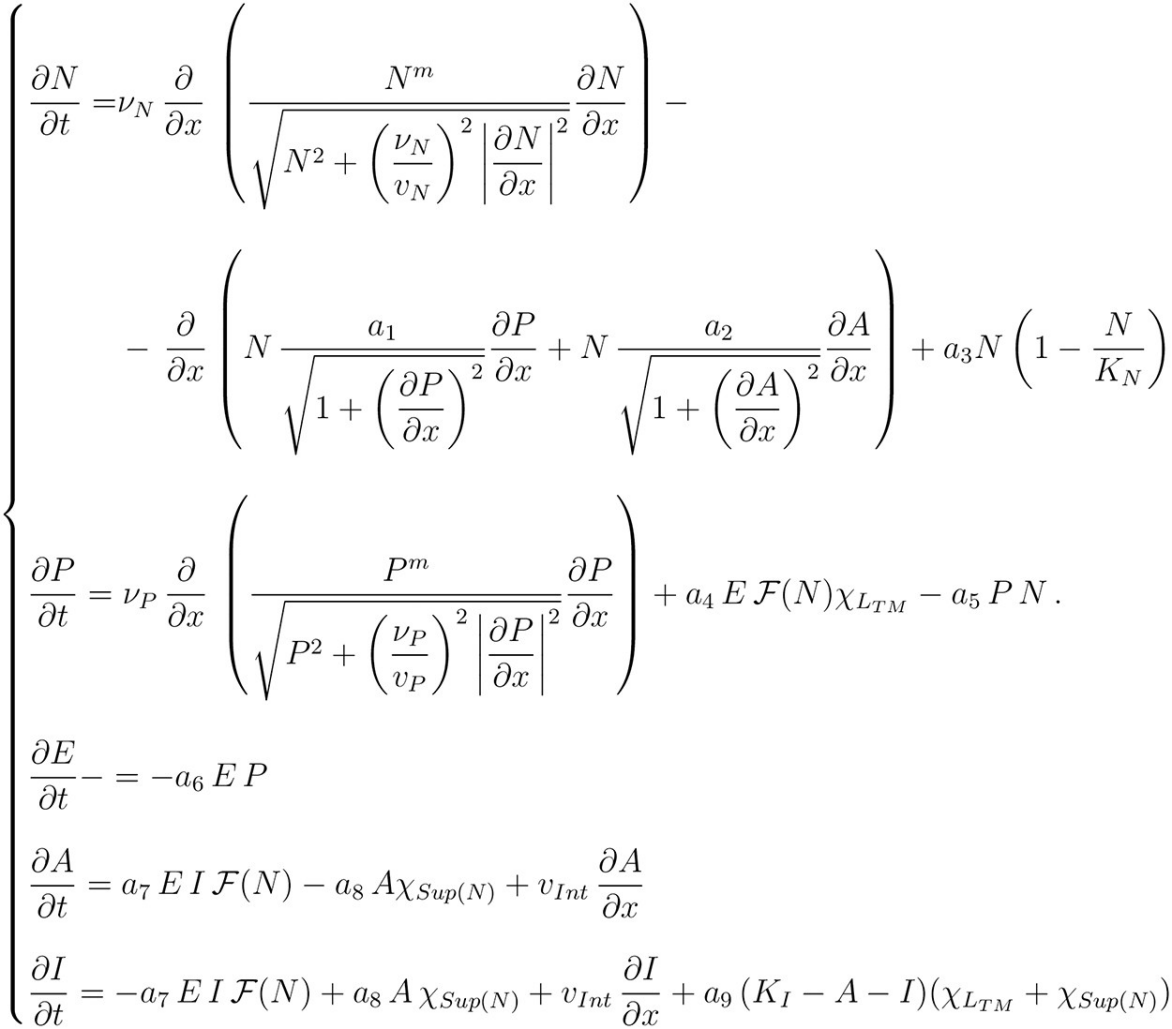
Whole system of differential equations describing the dynamics of the five species involved in the model.

From a mathematical viewpoint, we numerically solve the system given in Fig 6 with no flux boundary conditions.

**Fig 7:**
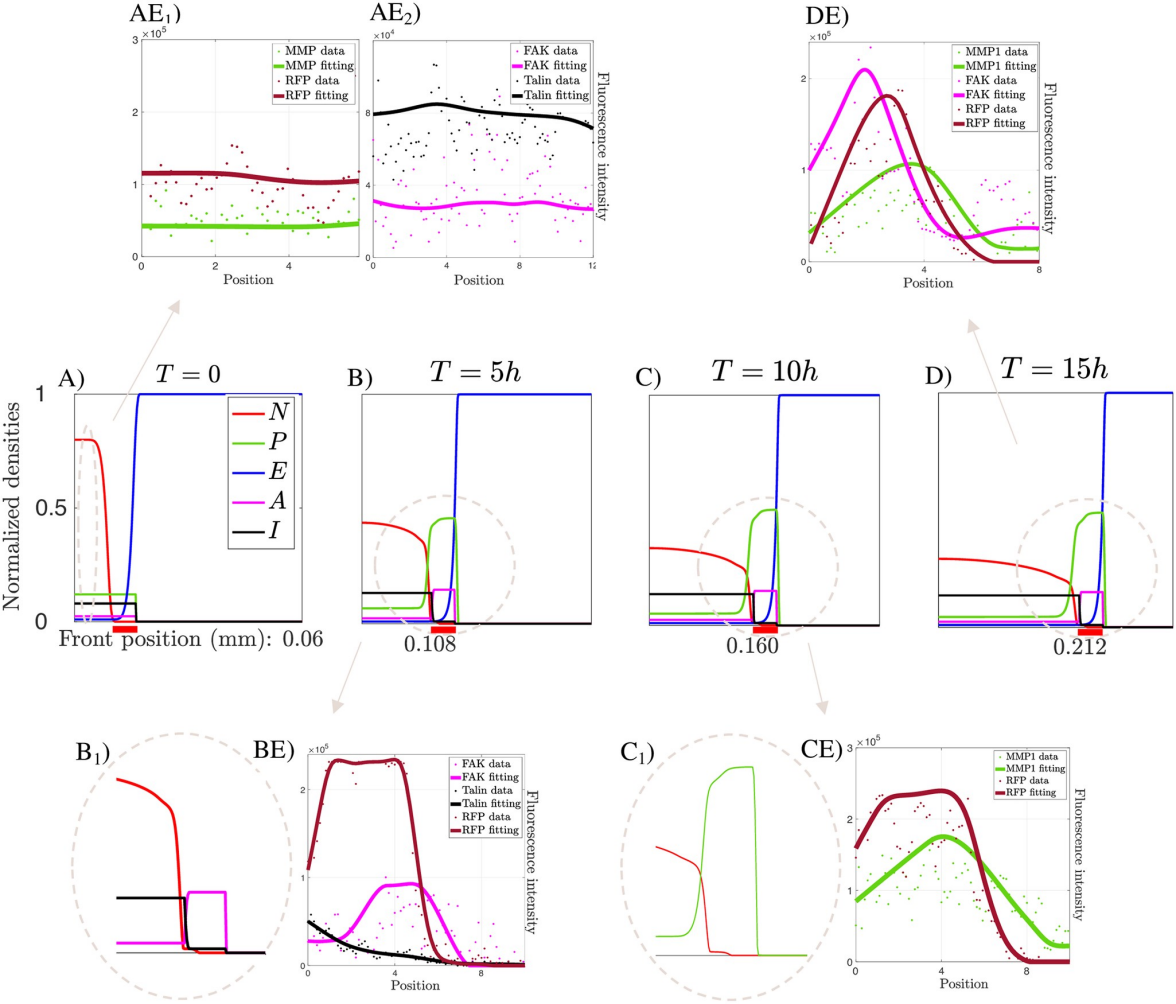
Modeling results. *A*), *B*), *C*) and *D*) are four snapshots of the numerical solution of the evolutionary model at the initial state, after 5, 10, and 15 hours, respectively. Images show the dynamics of tumor density (*N*), ECM (*E*), active (*A*) and inactive (*I*) integrins and MMP1s (*P*). The thick red line below the figures indicates the front area with the highest concentration of TMs. Images *AE*_*i*_), *BE*), *CE*) and *DE*) show the results of the analysis of the experimental data. In particular, peaks in the RFP distribution indicate areas with high tumor membrane density, i.e., the TM regions, while analogous peaks in the numerical simulations refer to high tumor density areas, i.e., the main tumor mass. The images *B*_1_) and *C*_1_) stand for a zoom of the indicated areas and they are correlated with the experimental data *BE* and *CE*, respectively. The parameters used for these simulations are listed in the S1 Table.

The results, represented in Fig 7, remarkably show how the model predicts that the region of greatest interest for the whole GB development process is the front, where TMs are located. The observed biological features correlate with the generation and progression of this critical tumor region, i.e., the front.

Fig 7*A* shows the initial condition of the system, and it is accompanied by the quantifications of the basal levels of MMP1s with respect to the tumor density (Fig 7*AE*_1_), and those of the active versus the inactive integrins (Fig 7*AE*_2_). In both cases, the measurements are taken from areas inside the bulk tumor and the resulting data are incorporated into the model.

Fig 7*B* and 7*C* show the time frames at 5 and 10 hours, respectively. The numerical simulations show that the model is capable of capture and predict the dynamics of the fronts and specific invasion profiles for each of the agents involved in the migration process. Precisely, Fig 7*B* and 7*C* illustrate how the model collects the exchange between active and inactive integrins at the beginning of the tumor front area, and how this corresponds to the experimental results (Fig 7*BE*). A plateau-like profile arises for *A*(*t*, *x*) in the TM region and, comparing Fig 7*B*_1_ with Fig 7*BE*, these numerical results show a good agreement with the experimental data. Moreover, the model outputs regarding the MMP1 front profile correlate with the results obtained experimentally, as shown in the comparison between Fig 7*C*_1_ and 7*CE*. MMP1 dynamics are characterized by an increased protein concentration along the TM region, indicating an enhanced tumor proteolytic activity, and by a steep profile ahead in the direction of tumor migration. Besides, we observe that the MMP1 external front is slightly shifted ahead with respect to the tumor front region. In fact, although MMP1s are produced along the protrusions, they are released in the extracellular space and spread in the areas around the tumor front. This behavior perfectly fits with our results.

Finally, in Fig 7*D* we show the situation after letting the model evolve for 15 hours. Fig 7*B*, 7*C* and 7*D* show that the invasion patterns of MMP1s and integrins are maintained over time. The experimental results in Fig 7*DE* provide the distributions of GB membrane, active integrins, and MMP1 markers at the same time and show their co-localization in the TM region. The results also show a displacement of the MMP1 distribution with respect to active integrin and tumor membrane distributions. The model outcomes coincide with the experimental data in Fig 7*DE*. The previous considerations about the relative positions of these three elements (GB membrane density, FAK, and MMP1 distribution) might be seen as an indicator of the direction of tumor migration.

As we developed in the S2 Text, the MMP1 activity modifies the porosity of the medium, facilitating the spread and, therefore, the transport and progression of the tumor mass. In particular, tumor cell advance velocity might not be constant, but dependent on the porosity of the medium. This dependency could produce modifications of the tumor front, as shown in the S3 Fig. Furthermore, depending on the concentration of MMP1s, even in the case of constant tumor cell velocity, the front of the tumor may lose regularity and sometimes break into two different fronts. Then, if the tumor growth is not homogeneous and there is a certain heterogeneity in the growth of the invasion fronts, modifications and splittings of the frontal structure might arise, in agreement with experimental evidence (see S4 Fig. for further details).

## Discussion

The location of the GB advancement front is essential to predict its evolutionary dynamics and it should help to design personalized therapies. In this work, we model and experimentally demonstrate that the active front of GB is a well-defined and consolidated structure. It harbors a considerable biochemical activity, defined by the protein ratio of integrins, proteases, and focal adhesions. Mathematically, the sharpness of the front results from a combination of the specific form of the flux term and the complex dynamical relationship between the agents involved in our system. This relationship also depends on the strength of the tactic mechanisms, due to integrin and protease activity and that drive GB migration. To ensure the robustness of the results, we analyze the possible influence of these tactic mechanisms on the tumor front profile as well as the effects of biomechanical changes in the porosity and stiffness of the tissue located closer to the TM area. The details of these additional studies are provided in the S1 Text and in the S3 and S4 Figs. These studies emphasize that, even if the tumor can break into separated masses, the profile steepness is preserved.

The mathematical model, based on the description of the specie dynamics involved in front progression, captures and reproduces the development of patterns that connect tumor, protease, and integrin evolution. These patterns characterize the dynamics of the GB front and have a predictive value on the directionality of the tumor migration. The mathematical model is based on a non-linear system of evolution equations (one for each of the involved agents) in which the mechanisms of production, chemotaxis, haptotaxis, and front dynamics compete with movement induced by the saturated flux in a porous media context. The contribution of mathematical models to tackle health problems is of great relevance. Usually, GB patients undergo surgery to remove the solid mass of the GB and a security resection margin, which can include a minimal amount of healthy tissue. However, the infiltrative nature of GB contributes to a possible re-appearance of the tumor. The results of the proposed multidisciplinary approach bring novelties that can help in the prediction of the behavior of the GB cells infiltrated in the brain. For instance, data from GB resection samples can be used to perform measurements of active/inactive integrins, MMP1s, and GB membrane density after surgery. In light of the model results on the relative location of these proteins, the outcomes of these measurements can contribute to predicting the invasive migratory behavior of GB cells, which may have relevant clinical consequences.

In conclusion, the feedback between experiments and modeling find common ground in our analysis to advance knowledge on the dynamics of the GB front. The results confirm that *Drosophila* model represents a suitable platform to analyze the molecular and cellular mechanisms implicated in GB progression. Our results point out the role of some of the main agents involved in the GB battlefront and the importance of incorporating this information into mathematical models in order to attempt to predict GB cell invasion dynamics.

## Methods

### Experimental procedures

#### Fly stocks

Flies were raised in standard fly food at 25C. Fly stocks from the Bloomington stock Centre: UAS-myr-RFP (BL7119), repo-Gal4 (BL7415), tub-gal80ts (BL7019). Vienna Drosophila Resource Center (VDRC): UAS-*mys* RNAi (BL33642), UAS-*rhea* RNAi (BL28950). UAS-dEGFRλ, UAS-PI3K92E (dp110CAAX) (A gift from R. Read).

#### *Drosophila* glioblastoma model

To reproduce glioblastoma in *Drosophila* we took advantage of the binary expression system Gal4/UAS [54]. We over-expressed constitutively active forms of EGFR and PI3K under the control of UAS sequences. To restrict this expression to glial cells, we used the specific enhancer repo (repo-Gal4>UAS-EGFRλ, UAS-dp110).

#### Immunostaining

We dissected third-instar larval brains in phosphate-buffered saline (PBS), fixed in 4% formaldehyde for 25min, washed in PBS + 0.3% Triton X-100 (PBT), and blocked in PBT + 5% BSA. Antibodies: mouse anti-MMP1 (DSHB 1:50), mouse anti-FAK (a gift from Isabel Guerrero, 1:50), rabbit anti-Talin (a gift from Isabel Guerrero, 1:50), Secondary antibodies: anti-mouse Alexa 488, 568, 647, anti-rabbit Alexa 488, 568, 647 (Thermofisher, 1:500).

#### Imaging

*Drosophila* brain images were mounted in Vectashield mounting media with DAPI (Vector Laboratories) and analyzed by Confocal microscopy (LEICA TCS SP5) with a 63x oil immersion objective and 3x magnification. Images were processed using Image J 1.52t.

#### Quantifications

FAK, MMP1, and Talin signals were determined from images taken at the same confocal settings. Pixel intensity was measured using the plot profile tool from Fiji 1.52t.

#### Survival

Animal survival is represented as the percentage of flies as compared to control siblings that reach adulthood upon GB induction (repo-Gal4>UAS-EGFRλ, UAS-dp110). n>100.

#### Numerical solution of the model

The whole system of partial differential equations (see Fig 6), coupled with no-flux boundary conditions, was numerically solved with a self-developed code in Matlab (MathWorks Inc., Natick, MA). For the spatial discretization, we considered the Galerkin method on a spatial grid of 500 points. Precisely, the flux-limited terms were discretized using an IMEX version of the Galerkin scheme. For the time discretization, an implicit Euler scheme was used for proteases and tumor equations, while a fourth-order Runge-Kutta method for the other involved populations over a total of 15 ⋅ 10^6^ time points.

#### Analysis of the biological data

For the analysis of the experimental data concerning the distributions of MMP1s, active and inactive integrins, we used a self-developed code in Matlab and the curve fitting toolbox, considering a weighted smoothing spline interpolation. In particular, for the analysis of MMP1 distribution, we analyzed 26 measurements on 8 different images taken from as many animals; then, for the distribution of Talin and FAK, we analyzed 14 measurements on 6 different images taken from as many animals; finally, for the combined distributions of FAK and MMP1, we analyzed 23 measurements on 13 different images taken from as many animals. The biological measurement data underlying the figures and the results presented in the text are provided in the S1 Data.

## Supporting information

S1 Text Mathematical model. (PDF)

S2 Text Parameter estimation. (PDF)

S3 Text Additional comments on the S1–S4 Figs. (PDF)

S1 Table Parameter estimation. (PDF)

S1 Fig Functional integrins are required for GB progression. Low magnification confocal images of *Drosophila* GB larvae brain in A) and *rhea* knockdown (UAS-*rhea* RNAi) in B), or *mys* knockdown (UAS-*mys* RNAi) in C). GB cell membrane is marked with myristoylated-RFP (red) and nuclei are marked with DAPI (blue). The images show that *rhea* or *mys* knockdown prevents the expansion of GB and rescues the lethality (percentage of survival in D).
(TIF)

S2 Fig Talin and FAK dynamics: Comparison between the inner GB mass and the GB front. Fluorescent confocal images of *Drosophila* 3rd instar larvae brain with GB marked in red (*A*_1_ and *B*_1_), and stained with anti-Talin (green in *A*_2_ and *B*_2_) and anti-FAK (magenta in *A*_3_ and *B*_3_). In *A*_4_ and *B*_4_, we show the quantification of the fluorescent signals and the graphical representation of the fluorescent intensity for GB, Talin and FAK signals along the white lines in *A*_1_-*A*_3_ and *B*_1_-*B*_3_ that indicate the location of the measurements. Dots represent the data and lines represent the fitting. The healthy tissue is the only component not marked in the immunostaining, thus it is represented by all the black regions in the images *A*_1_ and *B*_1_.
(TIF)

S3 FigEffects of porosity changes on tumor profile.*A*), *B*), and *C*) show the comparison between the tumor density profile in the case of flux saturated model with constant velocity *v*_*N*_ (in black) and with *v*_*N*_ = *v*_*N*_(*ϵ*) (in red) at the time steps referring to 3, 6 and 9 hours of tumor evolution, respectively. For this comparison we consider a tumor cell equation given by $\frac{\partial N}{\partial t}=-J_{\text{flux}-\text{sat}}\left(N\right)$. In *ϵ*/*v*), the profile of *v*_*N*_ = *v*_*N*_(*ϵ*) is shown. Specifically, accordingly with [8] (see Supplementary References in S2 Text), the minimum value for the velocity relates to a value of the porosity of 50%, while the maximum occurs around the value of 66%. The red curve shows how, as the velocity changes due to the ECM degradation process increase the medium porosity, cells closer to the front start moving faster than inner cells. This determines a heterogeneous modification of the invasion front, which slightly exceeds the homogenous front related to the constant velocity case. Eventually, the entire main tumor mass feels the changes in the velocity and a unique front is recovered. If there is heterogeneity in the growth of the front, the profile might not unify and a new front might emerge from this disturbance, as in the S4C Fig).(TIF)

S4 FigEffects of chemotaxis strength and heterogenous proliferation on tumor profile.*A*), *B*) and *C*) show the dynamics of the tumor profile after 5 hours in different cases. In *A*), the effect of changes in the chemotactic sensitivity *a*_1_ is presented. Since the proteolytic activity and, consequently, the concentration of MMP1 is enhanced in the front area, the stronger the parameter *a*_1_, the more evident the localization of the tactic effect. Tumor cells closer to the front acquire an increased overall velocity (due to both $J_{\text{flux}-\text{sat}}$ and $J_{\text{chemo}}$ fluxes) that leads to heterogenous fronts and, eventually, it might leads to a break of the tumor in two separated masses, as it can be observed in the experimental Fig *D*). In *D*), cell nuclei are marked in blue with DAPI, while GB cell membrane is marked with mystoylated-RFP in red. In particular, in *D*) higher intensity of the GB membrane marker indicates areas of tumor invasion. *B*) and *C*) show the comparison of our model with classical homogenous proliferation with the two possible models for heterogeneous proliferation, in the case of *a*_1_ = 0.005.(TIF)

S1 Data Excel file with the original biological data. (XLSX)
