# Supplementary material for: Modeling invasion patterns in the glioblastoma battlefield
Source: PLoS Comput Biol. 2021 Jan 29;17(1):e1008632. doi: 10.1371/journal.pcbi.1008632 (PMC7875342; doi:10.1371/journal.pcbi.1008632)
Supplement: S1 Text — (PDF) [file pcbi.1008632.s001.pdf]

Modeling invasion patterns in the glioblastoma battlefield

Martina Conte, Sergio Casas-Tintò, and Juan Soler

S1 Text: Mathematical model

Our mathematical model is made up of five coupled differential equations for the description of the concentrations of MMPs (P), active (A) and inactive (I) integrins, as well as for the densities of glioma cells (N) and ECM (E). All these variables are functions of time (t) and space (x). In particular, since our objective is the study of the tumor front dynamics, the differential equations for the different species, and their numerical simulations, are formulated in a 1D case, i.e., $(t, x) \in [0, T] \times \Omega$, with $T > 0$ and $\Omega = [0, b_\Omega] \subset \mathbb{R}$. We develop the model considering that tumor dynamics is characterized by a steep and well-defined front of invasion and increased tumor activity in the front region. The TM region (L_{TM}) and the function $\mathcal{F}(N)$, describing the heterogeneity in the tumor activity, have been characterized in equations (1) and (2), respectively, of the main text. Precisely, since in the main text we simply present an overview of the macroscopic equations for the different species and the involved mechanisms, here we provide a detailed description of the mathematical model formulation.

Glioma cell density $N(t, x)$ The evolution of glioma cell density is described using the following equation:

$$\frac{\partial N}{\partial t} = -\frac{\partial}{\partial x}\mathcal{J}_N(N, P, A) + \mathcal{P}_N(N)\,, \tag{1}$$

where $\mathcal{J}_N$ indicates the total flux of GB cells, and $\mathcal{P}_N$ refers to the proliferation term. In particular, the flux $\mathcal{J}_N$ includes three parts: $\mathcal{J}_{\text{flux-sat}}(N)$, $\mathcal{J}_{\text{chemo}}(N, P)$ and $\mathcal{J}_{\text{hapto}}(N, A)$, fluxes due to a flux-saturated mechanism, chemotaxis and haptotaxis, respectively.

The operator describing the flux-saturated mechanism is defined as

$$\mathcal{J}_{\text{flux-sat}}(N) := -\nu_N \frac{N^m}{\sqrt{N^2 + \left(\frac{\nu_N}{\upsilon_N}\right)^2 \left|\frac{\partial N}{\partial x}\right|^2}}\frac{\partial N}{\partial x}\,. \tag{2}$$

This type of operator was generally introduced with the so-called flux saturated equations in the form $\partial_t N = -\partial_x \mathcal{J}_{\text{flux-sat}}(N)$. These are equations in divergence form combining two non-linear diffusion mechanisms: the one of porous-media equations, as the exponent m is connected to the porosity of the medium [1]; the one for the flux saturation mechanism, which provides a flow that is saturated as long as the size of the gradients is large enough. The flux-saturated equations are characterized by a finite speed of propagation bounded by v_N, which limits the velocity of the propagation front and is incorporated in the model from the experimental data. Precisely, the velocity of the front depends on the internal pressure, and it is exactly v_N for $m = 1$, while it is limited by v_N for $m > 1$ [2,3]. m is also a parameter to be fitted from the experimental results. In this way, the solutions of the flux saturated equations preserve the characteristics of the initial data, in terms of compactness of the support and possible jump discontinuities, allowing for the emergence of steep invasion profiles. Finally, the parameter ν_N represents the viscosity of the medium with respect to the movement of tumor cells.

Models with saturated flows appear in the pioneer works of Wilson, Levermore, Rosenau, Agueh, Brenier, Andreu, Caselles, Mazon, among others, see [4–21], in the literature on Astrophysics, wave propagation in a medium and in optimal mass transport as an alternative to linear diffusion (see the survey [3] for a historical overview). In fact, the latter fails to precisely define a propagation front, since it is characterized by the phenomenon known as the infinite speed of propagation or instantaneous spreading. A model based on linear diffusion implies that GB cell would instantaneously reach and contaminate the whole brain tissue, to a greater or lesser extent, leading to the loss of the concept of tumor front and of the dynamics related to it. However, this is not commonly apparent. The capacity of tumor invasion and the possible response of the immune system makes necessary to focus our effort on controlling the battlefield. Therefore, the use of models based on saturated flux [2,22] allows us to locate and predict the dynamical evolution of the front.

The presence of MMP1s is necessary to degrade the ECM and allow the tumor cell to spread inside it. This process determines a chemotactic flux given by:

$$\mathcal{J}_{\text{chemo}}(N, P) = N \frac{a_1}{\sqrt{1 + \left(\dfrac{\partial P}{\partial x}\right)^2}} \frac{\partial P}{\partial x}\,, \tag{3}$$

where a_1 is the chemotactic sensitivity. This nonlinear form of the chemotactic flux reduces to the standard form under small protease gradients, while it saturates under large protease gradients.

The haptotactic flux $\mathcal{J}_{\text{hapto}}$, instead, is determined by the flux of active integrins that

mediate the attachment process between GB and ECM. It is described as

$$\mathcal{J}_{\text{hapto}}(N, A) = N \frac{a_2}{\sqrt{1 + \left(\frac{\partial A}{\partial x}\right)^2}} \frac{\partial A}{\partial x}, \tag{4}$$

where a_2 is the haptotactic sensitivity. Following the idea in [23], here the integrins produce an explicit effect on the direction of motion, describing the migration toward a gradient of recognized adhesion sites. By contrary with [23], we use a nonlinear form of the haptotactic gradient, analogous to the chemotactic one. Our novel approach is aimed at optimizing the influence on GB dynamics of the measurements of chemo or hapto forces along the trajectories, providing nonlinear terms in the corresponding Euler-Lagrange equations (3) and (4).

The proliferation term $\mathcal{P}_N(N)$ describes the tumor growth by means of a logistic term with proliferation rate a_3 and maximum carrying capacity K_N:

$$\mathcal{P}_N(N) = a_3 N \left(1 - \frac{N}{K_N}\right). \tag{5}$$

An improvement of this growth term should take into account the influence of morphogenic signaling pathways, such as Sonic Hedgehog (Shh) or Wnt (see [22,24–29] for further details). We will try to address this point in future works. The overall equation for the tumor population is given by equation (4) of the main text.

The combination of the flux-saturated mechanism and the chemo- and hapto-tactic fluxes might lead to different dynamics of the tumor profile, depending on the relative strength of the sensitivity parameters a_1 and a_2 and the tumor velocity v_N and viscosity ν_N. In the S4A Fig, we analyze how different values for the chemotactic sensitivity a_1 might provide a separation of the tumor front. The variation of this sensitivity confirms what was experimentally observed (see, for instance, S4D Fig) about the fact that the tumor cell profile and the tumor proliferation are not always homogenous, and tumor cells might assume a strong proliferative phenotype in specific regions. Based on the variation of the parameters, the model predicts two possibilities. Cells close to the inner front start to proliferate more in order to fill and reduce the distance between the two developed tumor fronts, as shown in the S4B Fig. Or, instead, cell close to the outer front proliferate more in order to create an autonomous front, determining tumor profiles similar to the one shown in the S4C Fig. The heterogeneous proliferation will be further investigated in future works since this phenomenon has a major impact and relevance in a higher spatial dimension case.

Metalloproteases concentration $P(t, x)$ We suppose that MMP1s are produced by tumor cells and released in the extracellular space to mediate the ECM degradation

process. Their dynamics are described as follow:

$$\frac{\partial P}{\partial t} = -\frac{\partial}{\partial x}\,\mathcal{J}_P(P) + \mathcal{P}_P(N,E) - \mathcal{D}_P(P,N)\,, \tag{6}$$

where $\mathcal{J}_P(P)$ is the MMP1 flux, $\mathcal{P}_P(N,E)$ the production term, and $\mathcal{D}_P(P,N)$ the degradation term.

The flux $\mathcal{J}_P(P)$ is described with the same flux-saturated mechanism used for the tumor cells, i.e.,

$$\mathcal{J}_P(P) = -\nu_P\,\frac{P^m}{\sqrt{P^2 + \left(\frac{\nu_P}{\upsilon_P}\right)^2 \left|\frac{\partial P}{\partial x}\right|^2}}\frac{\partial P}{\partial x}\,, \tag{7}$$

with υ_P velocity of MMP1s and ν_P viscosity related to MMP1 diffusion. The production term $\mathcal{P}_P(N,E)$ is given by:

$$\mathcal{P}_P(N,E) = a_4\,E\,\mathcal{F}(N)\chi_{L_{TM}}\,. \tag{8}$$

MMP1s production is localized on the protrusion region, whose characteristic function is given by $\chi_{L_{TM}}$, and is weighted using the tumor activity functional $\mathcal{F}(N)$. MMP1s are produced at rate a_4. Moreover, in the presence of ECM and once the tumor has moved forward, MMP1s degrade at rate a_5:

$$\mathcal{D}_P(P,N) = -a_5\,P\,N\,. \tag{9}$$

In particular, we assume that MMP1s degradation preserves a basal level of proteins in the inner regions of the tumor mass, estimated from the experiments. This basal level is mainly related to the normal cell proteolytic activity in the bulk tumor and is not directly aimed at sustaining the migration process. The overall equation for the protease population is given by equation (5) in the main text.

Extracellular matrix density $E(t,x)$ For the extracellular matrix we model the degradation process due to the proteolytic activity as:

$$\frac{\partial E}{\partial t} = -\mathcal{D}_E(E,P) \tag{10}$$

with degradation term $\mathcal{D}_E(E,P) = a_6\,E\,P$, and degradation rate a_6. Since, after the degradation process, some residual ECM material partially remains in the main tumor region, we include in the model a basal level of ECM inside the main tumor mass.

Active $A(t,x)$ and inactive $I(t,x)$ integrins concentration We divide the integrin family into two subpopulations, active and inactive integrins, defining as active integrins those receptors which are actively bound to the ligands of the extracellular matrix. This subfamily determines the gradient responsible for the haptotactic movement of tumor cells. The corresponding equation for the active integrins is made up of three different terms: integrin activation ($\mathcal{A}(E,I,N)$), integrin inactivation ($\mathcal{I}(A,N)$), and a flux term ($\mathcal{J}_A$)

$$\frac{\partial A}{\partial t} = \mathcal{A}(E,I,N) - \mathcal{I}(A,N) + \mathcal{J}_A\,. \tag{11}$$

We model the binding between GB cells and ECM through binary interactions of inactive integrins and ECM at rate a_7, weighting this process with the functional $\mathcal{F}_N$, i.e.,

$$\mathcal{A}(E,I,N) = a_7\, E\, I\, \mathcal{F}(N)\,. \tag{12}$$

Instead, once the tumor has moved forward, inactivation occurs at rate a_8 :

$$\mathcal{I}(A,N) = a_8\, A \chi_{Sup(N)}\,. \tag{13}$$

Here, $\chi_{Sup(N)}$ is the characteristic function of the support of N. Active integrins (as also inactive integrins), are subjected to a flux term describing the transport process due to the internal movement of GB cells. In fact, since integrin receptors are locate on the cell membrane, during the process of cell migration the receptors themselves are also transported:

$$\mathcal{J}_A = v_{Int}\,\frac{\partial A}{\partial x}\,. \tag{14}$$

The transport velocity v_{Int} is defined through a non-linear functional relationship that depends on the dynamics of N, i.e., $v_{Int} = v_{Int}[N]$. This functional describes the propagation rate of the support of N by means of its equation.

The dynamic interactions between active and inactive integrins are demonstrated by the attachment and detachment terms. Furthermore, inactive integrin dynamics are also characterized by a transport term ($\mathcal{J}_I$), analogous to $\mathcal{J}_A$, and a production process. In fact, we consider that new inactive integrins are created at rate a_9 and up to a maximum total level K_I, due to an exocytosis process ($\mathcal{P}_I$). Therefore, we define the following equation for inactive integrins dynamics:

$$\frac{\partial I}{\partial t} = -\mathcal{A}(E,I,N) + \mathcal{I}(A,N) + \mathcal{J}_I + \mathcal{P}_I\,, \tag{15}$$

where

$$\mathcal{P}_I = a_9\,(K_I - A - I)(\chi_{L_{TM}} + \chi_{Sup(N)}) \tag{16}$$

and

$$\mathcal{J}_I = v_{Int}\,\frac{\partial I}{\partial x}\,. \tag{17}$$

With this choice, the dynamics of the whole population of integrin receptors $(A + I)$ will be governed by a transport term, with velocity corresponding to the front propagation rate, and the exocytosis process.

Our interest in the role of integrins and proteases as leading mechanisms of tumor progression arises from a series of experiments that analyzed their activity and influence on tumor development. Integrin subunits $\alpha3$, $\alpha6$, and $\alpha7$, for instance, are expressed in stem cell-like GB cells, they localize especially in invading cells, and mediate invasion [30], growth and survival [31], as well as proliferation [32] and GB formation [33]. They have emerged as an enrichment marker for brain tumor malignancy [31] and as a promising anti-glioblastoma target. Several data show the ablation of the invasive capacity in cells knocked down for integrin $\alpha7$, in vitro and in vivo [31], the reduction of tumor formation for the functional knockdown of $\alpha6$ signaling pathway [33], and the improvement of patients survival in combined therapy with standard chemoradiation [30]. Similar results come also from several clinical studies combining a broad spectrum of MMP inhibitors (for instance marimastat, that inhibits MMP-1,-2,-7,-9) with temozolomide, showing promising beneficial effects on the increment of progression-free survival (see [34] and reference therein).

Supplementary References

1. Vázquez JL. The porous medium equation: mathematical theory. Oxford University Press; 2007.

2. Calvo J, Campos J, Caselles V, Sánchez O, Soler J. Pattern formation in a flux limited reactiondiffusion equation of porous media type. Inventiones mathematicae. 2016;206(1):57–108.

3. Calvo J, Campos J, Caselles V, Sánchez O, Soler J. Flux-saturated porous media equations and applications. EMS Surveys in Mathematical Sciences. 2015;2(1):131–218.

4. Levermore CD, Pomraning GC. A flux-limited diffusion theory. The Astrophysical Journal. 1981;248:321-334.

5. Mihalas D, Mihalas BW. Foundations of radiation hydrodynamics. Oxford University Press; 1984

6. Rosenau P. Tempered diffusion: A transport process with propagating fronts and inertial delay. Physical Review A. 1992;46(12):7371-7374.

7. Agueh M. Existence of solutions to degenerate parabolic equations via the Monge-Kantorovich theory. Ph.D. Thesis, Georgia Tech. 2001.

8. Brenier Y. Extended Monge–Kantorovich theory. In: Ambrosio L, Caffarelli LA, Brenier Y, Buttazzo G, Villani C, Salsa S editors. Optimal transportation and applications. Springer, Berlin, Heidelberg; 2003. pp. 91–121.

9. Andreu F, Calvo J, Mazón JM, Soler J. On a nonlinear flux-limited equation arising in the transport of morphogens. Journal of Differential Equations. 2012;252(10):5763–5813.

10. Andreu F, Caselles V, Mazón JM. The Cauchy problem for a strongly degenerate quasilinear equation. Journal of the European Mathematical Society. 2005;7(3):361–393.

11. Andreu F, Caselles V, Mazón JM, Moll S. Finite propagation speed for limited flux diffusion equations. Archive for rational mechanics and analysis. 2006;182(2):269–297.

12. Costa VC. Convergence of flux limited porous media diffusion equations to its classical counterpart. Annali della Scuola Normale Superiore di Pisa. Classe di scienze. 2015;14(2):481–505.

13. Caselles V. On the entropy conditions for some flux limited diffusion equations. Journal of Differential Equations. 2011;250(8):3311–2248.

14. Chertock A, Kurganov A, Rosenau P. Formation of discontinuities in flux-saturated degenerate parabolic equations. Nonlinearity. 2003;16(6):1875-1898.

15. Kurganov A, Rosenau P. On reaction processes with saturating diffusion. Nonlinearity. 2005;19(1):171.

16. McCann RJ, Puel M. Constructing a relativistic heat flow by transport time steps. Annales de l'IHP Analyse non linaire. 2009;26(6):2539–2580.

17. Bellomo N, Bellouquid A, Nieto J, Soler J. Multiscale biological tissue models and flux-limited chemotaxis for multicellular growing systems. Mathematical Models and Methods in Applied Sciences. 2010;20(07):1179–1207.

18. Arias M, Campos J, Soler J. Cross-diffusion and traveling waves in porous-media flux-saturated Keller–Segel models. Mathematical Models and Methods in Applied Sciences. 2018;28(11):2103–2129.

19. Calvo J, Mazón J, Soler J, Verbeni M. Qualitative properties of the solutions of a nonlinear flux-limited equation arising in the transport of morphogens. Mathematical Models and Methods in Applied Sciences. 2011;21(supp01):893–937.

20. Campos J, Guerrero P, Sánchez O, Soler J. On the analysis of traveling waves to a nonlinear flux limited reaction–diffusion equation. Annales de l'IHP Analyse non linaire. 2013;30(1):141–155.

21. Bellomo N, Bellouquid A, Tao Y, Winkler M. Toward a mathematical theory of Keller–Segel models of pattern formation in biological tissues. Mathematical Models and Methods in Applied Sciences. 2015;25(09):1663–1763.

22. Verbeni M, Sánchez O, Mollica E, Siegl–Cachedenier I, Carleton A, Guerrero I, et al. Morphogenetic action through flux-limited spreading. Physics of Life Reviews. 2013;10(4):457–475.

23. Mallet DG, Pettet GJ. A mathematical model of integrin-mediated haptotactic cell migration. Bulletin of mathematical biology. 2006;68(2):231.

24. González–Méndez L, Seijo–Barandiarán I, Guerrero I. Cytoneme-mediated cell-cell contacts for Hedgehog reception. Elife. 2017;6:e24045.

25. Cardozo MJ, Sánchez–Arrones L, Sandonis A, Sánchez–Camacho C, Gestri G, Wilson SW, et al. Cdon acts as a Hedgehog decoy receptor during proximal-distal patterning of the optic vesicle. Nature communications. 2014;5(1):1–13.

26. Sanders TA, Llagostera E, Barna M. Specialized filopodia direct long-range transport of SHH during vertebrate tissue patterning. Nature. 2013;497(7451):628–632.

27. Huang H, Liu S, Kornberg TB. Glutamate signaling at cytoneme synapses. Science. 2019;363(6430):948–955.

28. Kornberg TB, Gilboa L. Nanotubes in the niche. Nature. 2015;523(7560):292–294.

29. Portela M, Venkataramani V, Fahey–Lozano N, Seco E, Losada–Perez M, Winkler F, et al. Glioblastoma cells vampirize WNT from neurons and trigger a JNK/MMP signaling loop that enhances glioblastoma progression and neurodegeneration. PLoS biology. 2019;17(12):e3000545.

30. Nakada M, Nambu E, Furuyama N, Yoshida Y, Takino T, Hayashi Y, et al. Integrin $\alpha 3$ is overexpressed in glioma stem-like cells and promotes invasion. British journal of cancer. 2013;108(12):2516–2524.

31. Haas TL, Sciuto MR, Brunetto L, Valvo C, Signore M, Fiori ME, et al. Integrin $\alpha 7$ is a functional marker and potential therapeutic target in glioblastoma. Cell stem cell. 2017;21(1):35–50.

32. Guerrero PA, Tchaicha JH, Chen Z, Morales JE, McCarty N, Wang Q, et al. Glioblastoma stem cells exploit the $\alpha v \beta 8$ integrin-TGF$\beta 1$ signaling axis to drive tumor initiation and progression. Oncogene. 2017;36(47):6568–6580.

33. Lathia JD, Gallagher J, Heddleston JM, Wang J, Eyler CE, MacSwords J, et al. Integrin alpha 6 regulates glioblastoma stem cells. Cell stem cell. 2010;6(5):421–432.

34. Mentlein R, Hattermann K, Held–Feindt J. Lost in disruption: role of proteases in glioma invasion and progression. Biochimica et Biophysica Acta (BBA)-Reviews on Cancer. 2012;1825(2):178–185.
