# Supplementary material for: Modeling invasion patterns in the glioblastoma battlefield
Source: PLoS Comput Biol. 2021 Jan 29;17(1):e1008632. doi: 10.1371/journal.pcbi.1008632 (PMC7875342; doi:10.1371/journal.pcbi.1008632)
Supplement: S2 Text — (PDF) [file pcbi.1008632.s002.pdf]

Modeling invasion patterns in the glioblastoma battlefield

Martina Conte, Sergio Casas-Tintò, and Juan Soler

S2 Text: Parameters estimation

We consider a non-dimensionalized version of the system (shown in Fig 6, main text), scaling the five populations with respect to their carrying capacities (K_N, K_I) or to their typical concentrations/densities ($\hat{P}$, $\hat{E}$). S1 Table reports the values of the model parameters and the reference values used for the non-dimensionalization procedure. For the numerical simulations presented in Fig 7 (main text) we use $m = 1$.

Velocity and viscosity parameters The mean value reported in literature for glioma cell speed in human is 50 μm $\cdot$ h^{-1} [1]. Since our biological experiments are done in a *Drosophila* glioblastoma model, we deduce the value for the tumor propagation velocity in *Drosophila* considering the Stokes law. It expresses the frictional force, also called drag force, exerted on spherical objects with very small Reynolds numbers in a viscous fluid. As a consequence, this law deduces the relation between the object velocity v and radius R:

$$v = \frac{2(\rho_p - \rho_f)}{9\mu} \, g \, R^2 \,. \tag{1}$$

Here, ρ_p and ρ_f are the mass densities of the particles and the fluid, respectively, μ is the dynamic viscosity, and g the gravitational field strength [2]. Assuming an average size of $12 - 14\,\mu$m for human GB cells and $5\,\mu$m for GB cells in *Drosophila* (measurements taken from our experiments), we deduce the range $6.4 - 8.7\,\mu$m $\cdot$ h^{-1} for the parameter v_N. Considering the strong relation between MMP1s and GB cells, in terms of their respective locations and influences, and due to the lack of experimental data about protease propagation velocity in the brain, we assume $v_P = v_N$.

For the tumor viscosity ν_N, we refer to [3], where a similar description of the flux saturated mechanism is used for modeling the dynamics of the protein Shh. In [3], the authors consider Shh aggregates moving along a protrusion with speed $v_{Shh} = 1.3 \cdot 10^{-3}\,\mu$m $\cdot$ s^{-1} and kinematic viscosity $\nu_{Shh} = 5 \cdot 10^{-9}\,cm^2\cdot$ s^{-1}. Since in the description of the flux

saturated operator a key role is played by the ratio $\frac{\nu}{v}$, we use that $\frac{\nu_{Shh}}{v_{Shh}} = 0.4$ mm, and we deduce $\nu_N \in [0.256, 0.348] \cdot 10^{-2}$ mm$^2\cdot$ h^{-1}.

To deduce the protease viscosity ν_P, considering the similarity in size between MMP1 and Shh, we use the Einstein formula for diffusion of spherical particles [4]. For a spherical particle of radius R moving with uniform velocity in a continous fluid of viscosity μ, the frictional coefficient is given by $f_\tau = 6\,\pi\,\mu\,R$. Assuming that this applies also to spherical molecules, the kinematic viscosity is given by

$$\nu = \frac{K\,T}{6\,\pi\mu\,R} \tag{2}$$

with K Boltzmann constant and T temperature (in K). Considering that the radius of a vesicle containing Shh and moving along a cell protrusion satisfies $R_{Shh} \in [10, 100]$ nm, and using this information in equation (2), we get the following estimation

$$\frac{KT}{6\,\pi\mu} = \nu_{Shh}\,R_{Shh} = 0.18 \cdot [10^{-7}, 10^{-6}]\,\mathrm{mm}^3 \cdot \mathrm{h}^{-1}\,. \tag{3}$$

MMP1s have a radius of $R_P = 0.066 M_P^{1/3}$ [5], with R_P expressed in nm and the mass M_P in Da (Dalton). From [6], we know that $M_P \in [72, 92]$ kDa; therefore

$$R_P \in 0.66 \cdot [72^{\frac{1}{3}}, 92^{\frac{1}{3}}]\,\mathrm{nm} = [2.75, 2.98] \cdot 10^{-6}\,\mathrm{mm} \tag{4}$$

and

$$\nu_P = \frac{K\,T}{6\,\pi\,\mu\,R_P} = \frac{0.18 \cdot [10^{-7}, 10^{-6}]\,\mathrm{mm}^3 \cdot \mathrm{h}^{-1}}{[2.75, 2.98] \cdot 10^{-6}\,\mathrm{mm}} = [0.34, 0.36] \cdot [10^{-2}, 10^{-1}]\,\mathrm{mm}^2 \cdot \mathrm{h}^{-1}\,. \tag{5}$$

We set $\nu_P = 0.035\,\mathrm{mm}^2 \cdot \mathrm{h}^{-1}$.

As the proteases degrade the ECM, the porosity of the medium increases. This process can be modeled in two different ways. Using an equation for the degradation of the ECM (see equation (10) in S1 Text) and considering ECM influence on the dynamics of the protease and, consequently, on the spread of the tumor (which is the modeling approach we chose here). Or, an alternative way is to modify directly the porosity of the medium ϵ and to model its effect on the cell velocity. Several experiments [7,8] have shown the relation between the cell velocity and the size of the ECM pores. Especially in absence of proteolytic activity, too dense ECM does not allow cells to move inside it, since the pores are too narrow with respect the cell capability of squeezing and passing through it. At the same time, too large pores do not allow for cell migration either, since cell protrusions still need a certain amount of extracellular matrix around them in order to attach to it. Following the results of [8] (see, for instance, Fig 2.b inthere), we consider a variability range for $\epsilon \in [0.5, 0.75]$, and we define a law of variability for v_N, assuming an optimal

values of the tumor cell velocity for $\epsilon \sim 0.67$. Using an evolutionary law for ϵ analogous to the one proposed in [9]

$$\epsilon(t,x) = \epsilon_{max} - (\epsilon_{max} - \epsilon_0)\, e^{-\int_0^t a_6 P(\tau,x)\, d\tau} \tag{6}$$

with $\epsilon_0 = 0.54$ initial porosity value, and $\epsilon_{max} = 0.75$, we test our model for a scenario of non-constant v_N. Results are shown in the S3 Fig.

Carrying capacities The carrying capacity of tumor cells is estimated considering the mean diameter of a GB cell in *Drosophila* ($5\,\mu m$). This leads to an order of magnitude for the carrying capacity of $K_N \sim 10^6$ cells $\cdot$ mm^{-3}.

Instead, for the integrin capacity, considering that there are $\sim 10^5$ integrin receptors per cell [10], we estimate a maximum of $K_I \sim 10^{10}$ integrins $\cdot$ mm^{-3}.

Supplementary References

1. Milo R, Phillips R. Cell biology by the numbers. Garland Science; 2015.

2. Lamb H. Hydrodynamics. 4th ed. Cambridge university press; 1916.

3. Verbeni M, Sánchez O, Mollica E, Siegl–Cachedenier I, Carleton A, Guerrero I, et al. Morphogenetic action through flux-limited spreading. Physics of Life Reviews. 2013;10(4):457–475.

4. Edward JT. Molecular volumes and the Stokes-Einstein equation. Journal of Chemical Education. 1970;47(4):261.

5. Erickson HP. Size and shape of protein molecules at the nanometer level determined by sedimentation, gel filtration, and electron microscopy. Biological procedures online. 2009;11(1):32.

6. Mercapide J, Lopez De Cicco R, Castresana JS, Klein-Szanto AJ. Stromelysin1/matrix metalloproteinase3 (MMP3) expression accounts for invasive properties of human astrocytoma cell lines. International journal of cancer. 2003;106(5):676–682.

7. Wolf K, Te Lindert M, Krause M, Alexander S, Te Riet J, Willis AL, et al. Physical limits of cell migration: control by ECM space and nuclear deformation and tuning by proteolysis and traction force. Journal of Cell Biology. 2013;201(7):1069–1084.

8. Zaman MH, Trapani LM, Sieminski AL, MacKellar D, Gong H, Kamm RD. Migration of tumor cells in 3D matrices is governed by matrix stiffness along with cell-matrix adhesion and proteolysis. Proceedings of the National Academy of Sciences. 2006;103(29):10889–10894.

9. Niibori Y, Usui H, Chida T. Double porosity model to describe both permeability change and dissolution processes. Mechanical Engineering Journal. 2015;2(5):15–00210.

10. Belkin AM, Tsurupa G, Zemskov E, Veklich Y, Weisel JW, Medved L. Transglutaminase-mediated oligomerization of the fibrin (ogen) C domains promotes integrin-dependent cell adhesion and signaling. Blood. 2005;105(9):3561–3568.
