# Supplementary material for: Modeling invasion patterns in the glioblastoma battlefield
Source: PLoS Comput Biol. 2021 Jan 29;17(1):e1008632. doi: 10.1371/journal.pcbi.1008632 (PMC7875342; doi:10.1371/journal.pcbi.1008632)
Supplement: S3 Text — (PDF) [file pcbi.1008632.s003.pdf]

Modeling invasion patterns in the glioblastoma battlefield

Martina Conte, Sergio Casas-Tintò, and Juan Soler

S3 Text: Additional comments on the S1-S4 Figs.

To validate the functional contribution of integrins to GB progression we use specific RNAi constructs to knockdown *myospheroid (mys)* or *rhea*, two key players for integrin function. *mys* encodes a β subunit of the integrin dimer that acts as adhesion/signaling protein, regulating cellular adhesion and migration. Besides, *rhea* encodes *Drosophila* Talin, a large adaptor protein essential for adhesive functions of integrins. Confocal images of *Drosophila* brains with GB show large brains with expanded GB cell membrane (red S1A Fig). Upon *mys* or *rhea* knockdown, GB expansion is impaired (S1B and S1C Figs) and the lethality caused by GB is partially rescued (S1D Fig). These results suggest that GB cells require intact integrin function to progress, expand and cause premature death.

We quantified Talin and FAK immunostaining signals in confocal microscopy GB images to validate the inverse relation of Talin and FAK in inner GB mass and at GB front. The results show that the inner region of the GB mass (S2A_1 Fig) has higher Talin protein levels (S2A_2 Fig) and lower FAK signal (S2A_3 Fig), as represented in S2A_4 Fig. In line with our previous results, the relative concentration of Talin and FAK is inverted in the front region of GB samples (S2B_1 Fig). The results, in fact, indicate that Talin concentration drops significantly (S2B_2 and S2B_4 Figs) and correlates with an increase of FAK signal at GB front (S2B_3 and S2B_4 Figs). These observations suggest that Talin and FAK maintain an inverse correlation, and they are indicators of the migratory status of GB cells.
