# Supplementary material for: Modeling invasion patterns in the glioblastoma battlefield
Source: PLoS Comput Biol. 2021 Jan 29;17(1):e1008632. doi: 10.1371/journal.pcbi.1008632 (PMC7875342; doi:10.1371/journal.pcbi.1008632)
Supplement: S1 Table — (PDF) [file pcbi.1008632.s004.pdf]

Modeling invasion patterns in the glioblastoma battlefield

Martina Conte, Sergio Casas-Tintò, and Juan Soler

Parameter	Description	Value (units)	Source
ν_N	tumor viscosity	$[0.256, 0.348] \cdot 10^{-2}$ ($mm^2 \cdot h^{-1}$)<br>used value: 0.00348	see S2 Text
v_N	tumor velocity	$[0.64, 0.87] \cdot 10^{-2}$ ($mm \cdot h^{-1}$)<br>used value: 0.0087	see S2 Text
a_1	chemotactic sensitivity	0.001 ($mm^2 \cdot h^{-1}$)	[1]
a_2	haptotactic sensitivity	0.0036 ($mm^2 \cdot h^{-1}$)	[1]
a_3	tumor proliferation rate	0.0345 (h^{-1})	[2]
K_N	tumor carrying capacity	$\sim 10^6$ ($cells \cdot mm^{-3}$)	see S2 Text
ν_P	MMPs viscosity	0.035 ($mm^2 \cdot h^{-1}$)	see S2 Text
v_P	MMPs velocity	$[0.64, 0.87] \cdot 10^{-2}$ ($mm \cdot h^{-1}$)<br>used value: 0.0087	see S2 Text
a_4	MMPs production rate	$[3.6, 180]$ (h^{-1})<br>used value: 3.6	[3]
a_5	MMPs degradation rate	$[0.18, 18]$ (h^{-1})<br>used value: 18	[4]
a_6	ECM degradation rate	~ 4.5 (h^{-1})	[5,6]
a_7	integrin activation rate	$[1, 3] \cdot 36$ (h^{-1})<br>used value: 108	[7]
a_8	integrin inactivation rate	36 (h^{-1})	[7]
a_9	integrin exocytosis rate	$[0.36, 36]$ (h^{-1})<br>used value: 0.72	[8]
K_I	integrins maximum capacity	$\sim 10^{10}$ ($integrins \cdot mm^{-3}$)	see S2 Text
$\hat{E}$	ECM reference value	10^{-3} ($mg \cdot mm^{-3}$)	[4]
$\hat{P}$	protease reference value	10^{-7} ($mg \cdot mm^{-3}$)	[4]

Table S1: Parameter estimation.

Supplementary References

1. Kim Y, Lee W, Jeon H, Lim S, Roh S, Lee D, et al. The Role of Microenvironment in Regulation of Cell Infiltration in Glioblastoma. In: Stolarska M, Tarfulea NE, editors. Cell Movement. Birkhäuser, Cham; 2018. pp. 27–60.

2. Mercapide J, Lopez De Cicco R, Castresana JS, Klein-Szanto AJ. Stromelysin1/matrix metalloproteinase3 (MMP3) expression accounts for invasive properties of human astrocytoma cell lines. International journal of cancer. 2003;106(5):676–682.

3. Kumar S, Das A, Barai A, Sen S. MMP secretion rate and inter-invadopodia spacing collectively govern cancer invasiveness. Biophysical journal. 2018;114(3):650–662.

4. Kim Y, Roh S, Lawler S, Friedman A. miR451 and AMPK mutual antagonism in glioma cell migration and proliferation: a mathematical model. PloS one. 2011;6(12):e28293.

5. Saitou T, Rouzimaimaiti M, Koshikawa N, Seiki M, Ichikawa K, Suzuki T. Mathematical modeling of invadopodia formation. Journal of theoretical biology. 2012;298:138–146.

6. Olson MW, Gervasi DC, Mobashery S, Fridman R. Kinetic analysis of the binding of human matrix metalloproteinase-2 and-9 to tissue inhibitor of metalloproteinase (TIMP)-1 and TIMP-2. Journal of Biological Chemistry. 1997;272(47):29975–29983.

7. Lauffenburger DA, Linderman JJ. Receptors: models for binding, trafficking, and signaling. Oxford University Press on Demand; 1996.

8. Dickinson RB, Tranquillo RT. A stochastic model for adhesion-mediated cell random motility and haptotaxis. Journal of mathematical biology. 1993;31(6):563–600.
