# Supplementary figures and images for: Modeling invasion patterns in the glioblastoma battlefield
Source: PLoS Comput Biol. 2021 Jan 29;17(1):e1008632. doi: 10.1371/journal.pcbi.1008632 (PMC7875342; doi:10.1371/journal.pcbi.1008632)

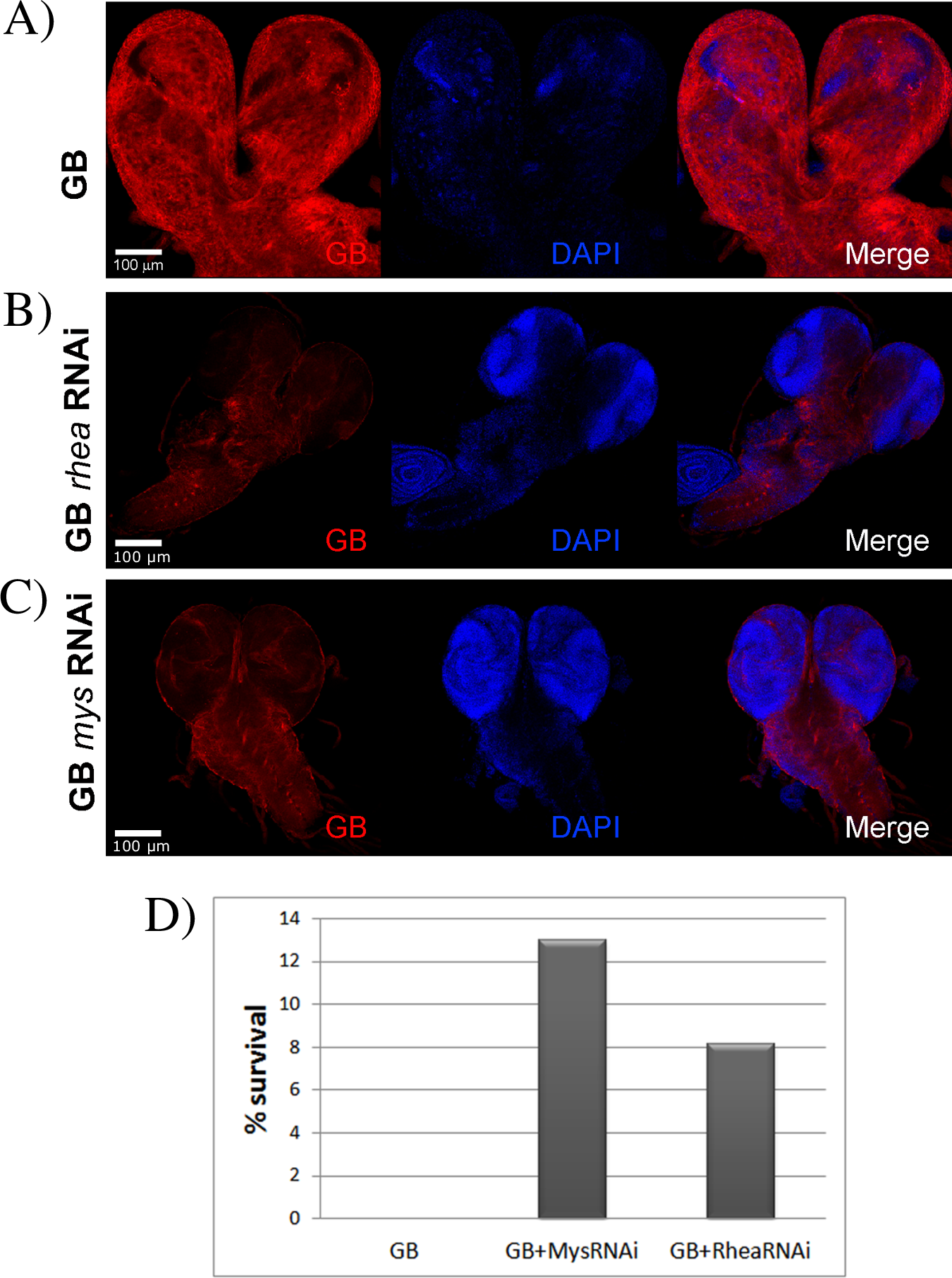

Supplement: S1 Fig — Low magnification confocal images of Drosophila GB larvae brain in A) and rhea knockdown (UAS-rhea RNAi) in B), or mys knockdown (UAS-mys RNAi) in C). GB cell membrane is marked with myristoylated-RFP (red) and nuclei are marked with DAPI (blue). The images show that rhea or mys knockdown prevents the expansion of GB and rescues the lethality (percentage of survival in D). (TIF) [file pcbi.1008632.s005.tif]

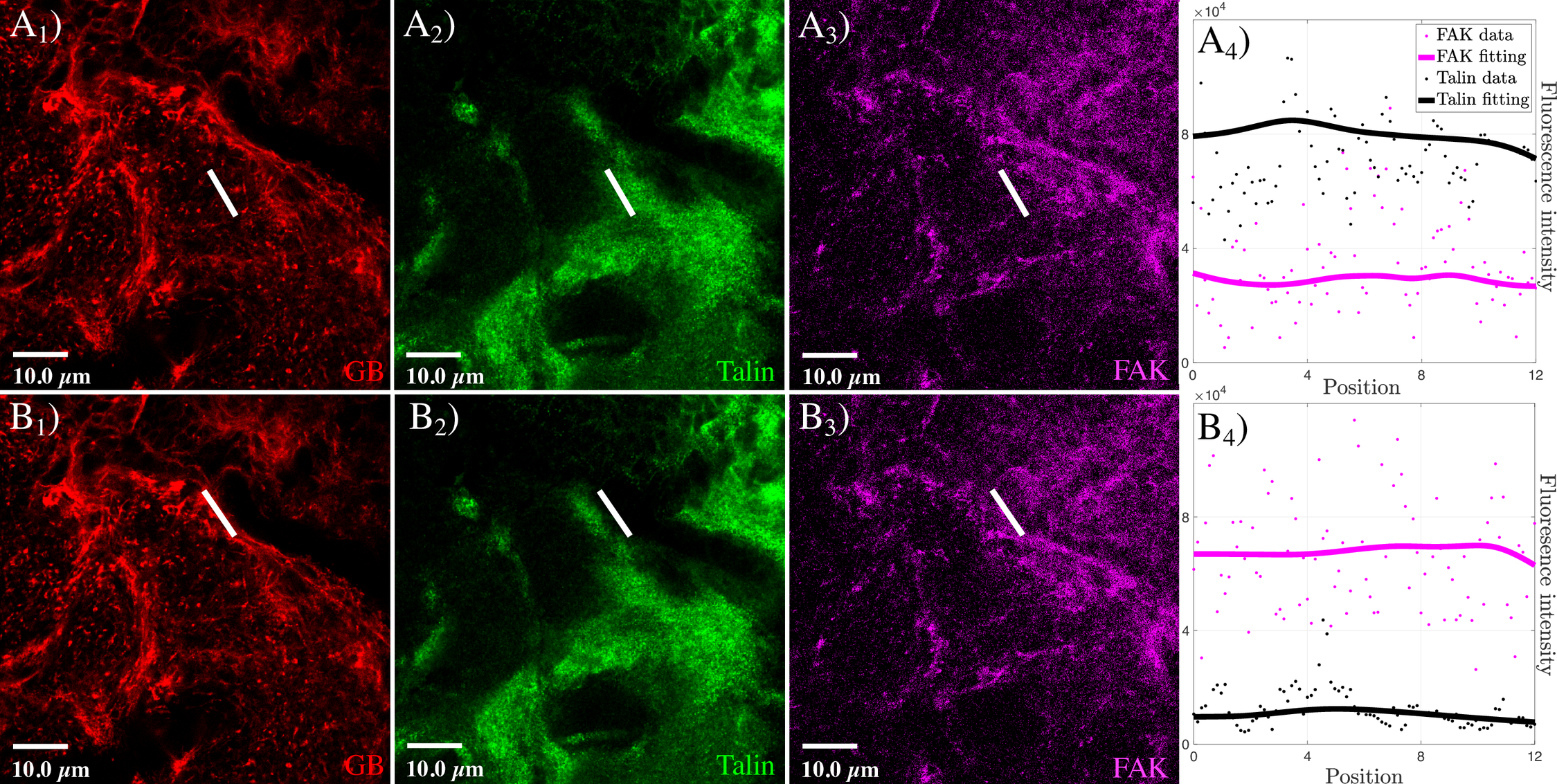

Supplement: S2 Fig — Fluorescent confocal images of Drosophila 3rd instar larvae brain with GB marked in red (A1 and B1), and stained with anti-Talin (green in A2 and B2) and anti-FAK (magenta in A3 and B3). In A4 and B4, we show the quantification of the fluorescent signals and the graphical representation of the fluorescent intensity for GB, Talin and FAK signals along the white lines in A1-A3 and B1-B3 that indicate the location of the measurements. Dots represent the data and lines represent the fitting. The healthy tissue is the only component not marked in the immunostaining, thus it is represented by all the black regions in the images A1 and B1. (TIF) [file pcbi.1008632.s006.tif]

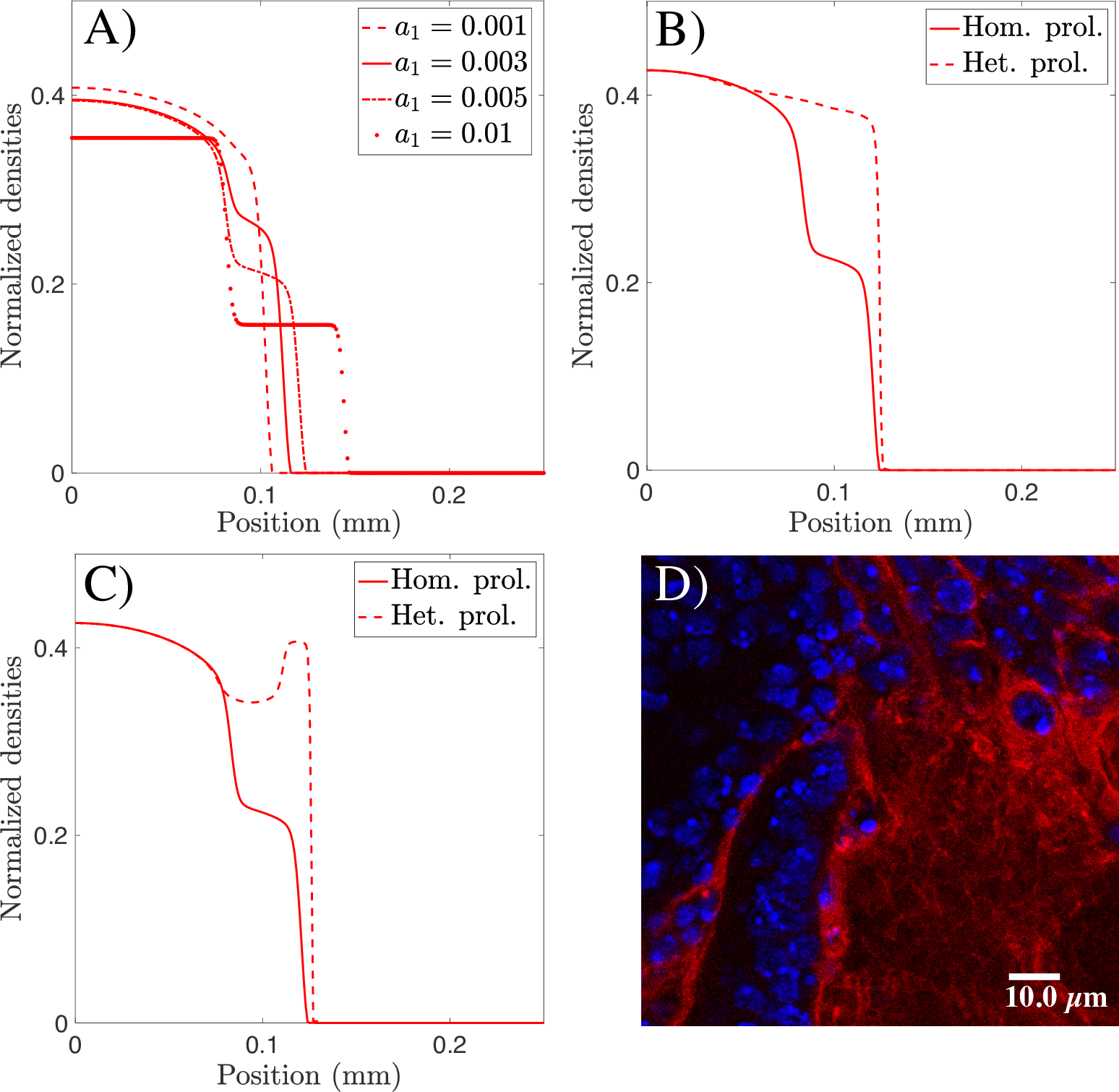

Supplement: S3 Fig — A), B), and C) show the comparison between the tumor density profile in the case of flux saturated model with constant velocity vN (in black) and with vN = vN(ϵ) (in red) at the time steps referring to 3, 6 and 9 hours of tumor evolution, respectively. For this comparison we consider a tumor cell equation given by ∂N∂t=-Jflux-sat(N). In ϵ/v), the profile of vN = vN(ϵ) is shown. Specifically, accordingly with [8] (see Supplementary References in S2 Text), the minimum value for the velocity relates to a value of the porosity of 50%, while the maximum occurs around the value of 66%. The red curve shows how, as the velocity changes due to the ECM degradation process increase the medium porosity, cells closer to the front start moving faster than inner cells. This determines a heterogeneous modification of the invasion front, which slightly exceeds the homogenous front related to the constant velocity case. Eventually, the entire main tumor mass feels the changes in the velocity and a unique front is recovered. If there is heterogeneity in the growth of the front, the profile might not unify and a new front might emerge from this disturbance, as in the S4C Fig). (TIF) [file pcbi.1008632.s007.tif]

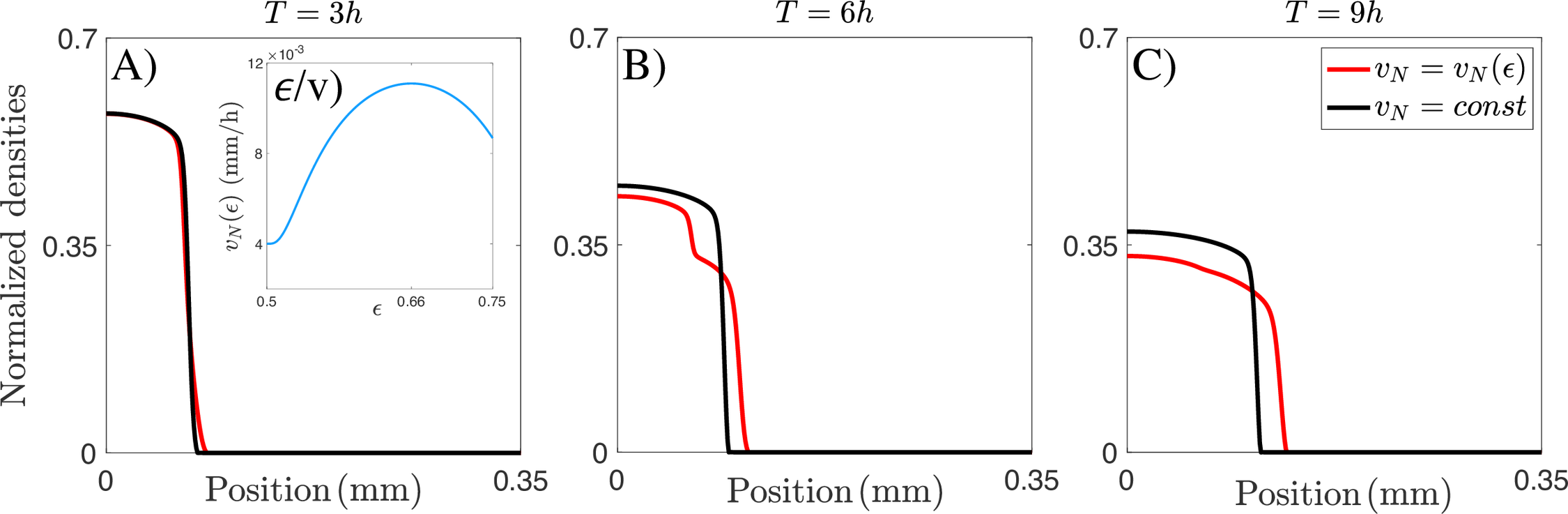

Supplement: S4 Fig — A), B) and C) show the dynamics of the tumor profile after 5 hours in different cases. In A), the effect of changes in the chemotactic sensitivity a1 is presented. Since the proteolytic activity and, consequently, the concentration of MMP1 is enhanced in the front area, the stronger the parameter a1, the more evident the localization of the tactic effect. Tumor cells closer to the front acquire an increased overall velocity (due to both Jflux-sat and Jchemo fluxes) that leads to heterogenous fronts and, eventually, it might leads to a break of the tumor in two separated masses, as it can be observed in the experimental Fig D). In D), cell nuclei are marked in blue with DAPI, while GB cell membrane is marked with mystoylated-RFP in red. In particular, in D) higher intensity of the GB membrane marker indicates areas of tumor invasion. B) and C) show the comparison of our model with classical homogenous proliferation with the two possible models for heterogeneous proliferation, in the case of a1 = 0.005. (TIF) [file pcbi.1008632.s008.tif]
